# LPAR_1_ regulates enteric nervous system function through glial signaling and contributes to chronic intestinal pseudo-obstruction

**DOI:** 10.1172/JCI149464

**Published:** 2022-02-15

**Authors:** Mohammad M. Ahmadzai, Jonathon L. McClain, Christine Dharshika, Luisa Seguella, Fiorella Giancola, Roberto De Giorgio, Brian D. Gulbransen

**Affiliations:** 1 Department of Physiology, Neuroscience Program,; 2 College of Osteopathic Medicine, and; 3 College of Human Medicine, Michigan State University, East Lansing, Michigan, USA.; 4 Department of Physiology and Pharmacology “V. Erspamer”, Sapienza University of Rome, Rome, Italy.; 5 Department of Translational Medicine, University of Ferrara, Ferrara, Italy.; 6 St. Orsola-Malpighi Hospital, Bologna, Italy.

**Keywords:** Gastroenterology, Neuroscience, Calcium signaling, G protein&ndash;coupled receptors, Homeostasis

## Abstract

Gastrointestinal motility disorders involve alterations to the structure and/or function of the enteric nervous system (ENS) but the causal mechanisms remain unresolved in most cases. Homeostasis and disease in the ENS are processes that are regulated by enteric glia. Signaling mediated through type I lysophosphatidic acid receptors (LPAR_1_) has recently emerged as an important mechanism that contributes to disease, in part, through effects on peripheral glial survival and function. Enteric glia express LPAR_1_ but its role in ENS function and motility disorders is unknown. We used a combination of genetic, immunohistochemical, calcium imaging, and in vivo pharmacological approaches to investigate the role of LPAR_1_ in enteric glia. LPAR_1_ was enriched in enteric glia in mice and humans and LPA stimulated intracellular calcium responses in enteric glia, subsequently recruiting activity in a subpopulation of myenteric neurons. Blocking LPAR_1_ in vivo with AM966 attenuated gastrointestinal motility in mice and produced marked enteric neuro- and gliopathy. Samples from humans with chronic intestinal pseudo-obstruction (CIPO), a severe motility disorder, showed reduced glial LPAR_1_ expression in the colon and ileum. These data suggest that enteric glial LPAR_1_ signaling regulates gastrointestinal motility through enteric glia and could contribute to severe motility disorders in humans such as CIPO.

## Graphical abstract

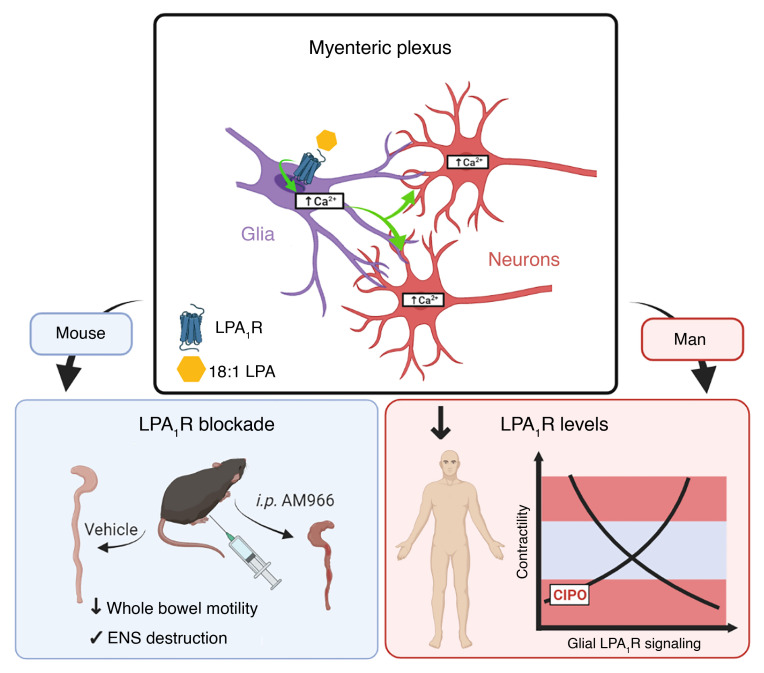

## Introduction

Disturbances of gastrointestinal (GI) motility are common in humans and contribute to major economic and personal burdens worldwide (1). GI motility disorders such as gastroparesis and intestinal pseudo-obstruction are specifically classified by persistent or recurrent dysmotility, although abnormal GI motility is also common in disorders of the gut-brain axis, such as irritable bowel syndrome (2). Chronic intestinal pseudo-obstruction (CIPO) is an example of a severe motility disorder and is characterized by recurrent episodes of intestinal subocclusion that occur in the absence of mechanical blockade (3). Systemic diseases of the neuro-musculature controlling gut motility account for secondary forms of CIPO, though most cases are idiopathic. In these patients, histological examination may reveal nonspecific neuropathic (4), myopathic, or mesenchymopathic (5) alterations of the gut wall. Clinically, CIPO is associated with substantial morbidity and poor quality of life owing to a lack of effective treatment options (6), which range from the use of prokinetic agents (7), to gut transplantation (8) in dire cases. These diagnostic and therapeutic shortcomings highlight major gaps in the management of motility disorders such as CIPO that result from a poor understanding of their pathophysiological basis.

GI motility is controlled by the enteric nervous system (ENS), an autonomous division of the peripheral nervous system embedded within the gut wall (9). Intrinsic enteric circuitry composed of sensory, inter-, and motor neurons is sufficient to coordinate patterns of gastrointestinal movement with minimal input from the brain and spinal cord (10). Thus, changes to the function and/or survival of enteric neurons is considered an important mechanism in GI motility disorders (11), and enteric neuropathy is a defining histological feature of neuropathic forms of CIPO (4). Although immune cell infiltrates are observed in the ENS of some of these patients (12), neuronal degeneration can occur without overt signs of tissue inflammation. This suggests that a broader mechanism common to both pathways may ultimately drive CIPO pathogenesis.

Disease in the nervous system is often viewed as a disruption of homeostatic processes controlled by glia (13). In the ENS, these roles are fulfilled by a unique population of peripheral neuroglia called enteric glia (14). Enteric glia regulate GI motility through bidirectional communication with enteric neurons (15–21) and contribute to neuroinflammation in disease (22–26). These attributes suggest that glial mechanisms could contribute to GI motility disorders and this is supported by data from animal models which show that perturbing glial functions alters motility (15, 18, 19), promotes neurodegeneration during acute colitis (22, 23), and influences immune responses (25, 27–29). However, specific mechanisms by which enteric glia might contribute to motility disorders remain mostly uncharacterized.

Lysophosphatidic acid (LPA) signaling through type I LPA receptors (LPAR_1_) has emerged as an important regulator of glial function and survival in the periphery (30–32) and the brain (33, 34). LPA levels are also elevated during disease and contribute to neuroplasticity and gut pathophysiology (35–38). Enteric glia are sensitive to LPA in cell culture experiments (17, 39) but whether this characteristic is maintained in vivo and if so, what functional significance it might have, is unknown. We hypothesized that LPA might regulate ENS function through effects on glia and that disturbing this process contributes to motility disorders. We tested our hypothesis in experiments that incorporated cellular imaging, organ physiology, whole animal models, and human samples. The data show that enteric glia express high levels of LPAR_1_ in mice and humans and that stimulating glial LPAR_1_ affects myenteric neuron excitability and gut motility. Further, we show that glial LPAR_1_ expression is deficient in samples from humans with CIPO and that blocking LPAR_1_ produces profound disturbances of gut motility and ENS structure in mice. Together, these data highlight LPA signaling as an important mechanism that regulates myenteric motor circuits through effects on glia and suggest that alterations to this process could contribute to motility disorders such as CIPO.

## Results

### LPAR_1_ expression is conserved in human and murine enteric glia.

**Figure 1:**
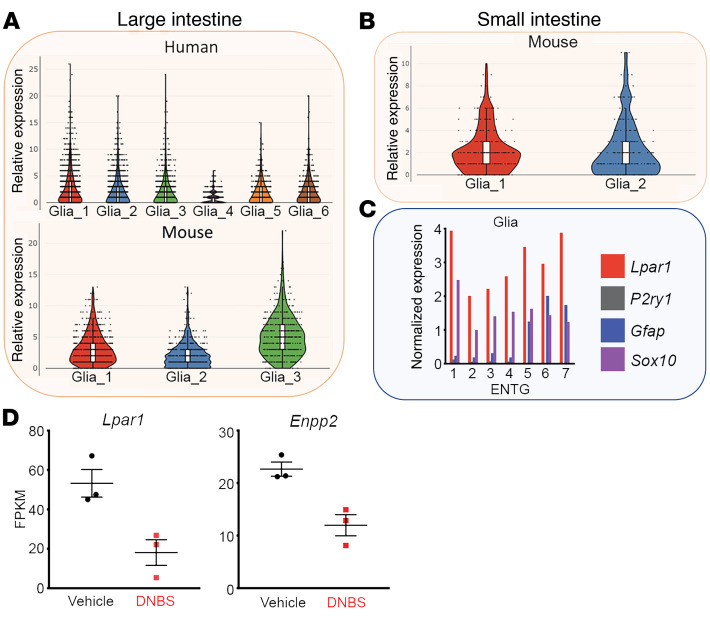
*LPAR1/Lpar1* gene expression in mouse and human enteric glia. (**A** and **B**) Single-cell RNA sequencing data sourced from ref. 40, showing *LPAR1/Lpar1* expression among enteric glial subtypes in the human (**A**, top) and mouse (**A**, bottom) large intestine and mouse small intestine (**B**). (**C**) Single-cell RNA sequencing data sourced from ref. 39, showing normalized glial *Lpar1* expression in comparison to *Sox10, Gfap*, *P2ry1*. Expression in enteric neurons is shown in Supplemental Figure 1. (**D**) Bulk RNA sequencing data sourced from ref. 22, showing that colonic glia exhibit reduced expression of *Lpar1* (left) and *Enpp2* (right) during acute DNBS-mediated colitis. *Enpp2* is an ecto-enzyme that catalyzes production of lysophospholipids acting at LPAR_1_. FPKM, fragments per kilobase of transcript per million mapped reads.

We began by assessing the potential breadth of LPA signaling in the human and mouse ENS by screening publicly available databases that capture ENS gene expression with single-cell resolution (40, 41). Nearly all enteric glia robustly express *LPAR1* in the human large intestine (Figure 1A) and *Lpar1* in the mouse small and large intestines (Figure 1, A–C). Bulk RNA sequencing of the colonic enteric glial translatome (22) also demonstrates high *Lpar1* expression in enteric glia (Figure 1D). This expression was complemented by robust expression of the gene for the LPA-generating enzyme, ectonucleotide pyrophosphatase type 2 (*Enpp2*; ref. 42). *Lpar1* and *Enpp2* gene expression were reduced in the mouse during the acute phase of dinitrobenzenesulfonic acid–induced (DNBS-induced) colitis, supporting a possible role for altered glial LPAR_1_ signaling in the pathogenesis of dysmotility following an inflammatory insult. These data are in line with prior work that reported LPA responsiveness in cultured enteric glia from neonatal guinea pigs and mice (17, 39) and extend those observations to suggest that glia remain LPA-responsive in vivo. Importantly, *LPAR1/Lpar1* gene expression was null or lowly expressed in nearly all enteric neuron subtypes in both species, indicating that its ENS roles are likely glial-mediated (Supplemental Figure 1, A–C; supplemental material available online with this article; https://doi.org/10.1172/JCI149464DS1). This is also consistent with prior work showing that LPA does not have direct effects on enteric neuron excitability (17). It should be noted that of the 6 LPAR subtypes (42), the remaining LPAR_2–6_ subtypes were undetectable in both human and murine enteric glia (not shown).

**Figure 2:**
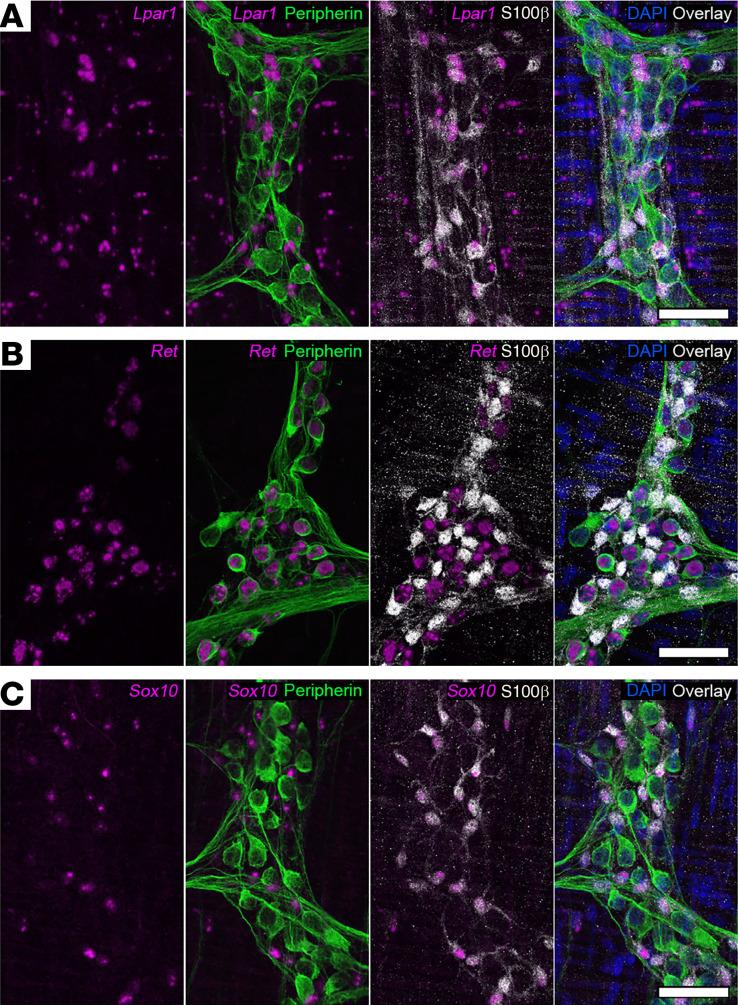
Distribution of *Lpar1* mRNA in the mouse colon myenteric plexus. (**A**) Combined in situ hybridization (RNAscope) and immunofluorescence showing *Lpar1* mRNA (magenta) expression in combination with markers of neurons (peripherin, green) and glia (S100β, gray). *Lpar1* is primarily expressed by S100β-positive glia in the myenteric plexus. (**B** and **C**) Validation of RNAscope protocol sensitivity and specificity in the murine ENS. RNAscope probes for *Ret* (**B**, leftmost panel, magenta) and *Sox10* (**C**, leftmost panel, magenta) demonstrate neuronal (peripherin, green) and glial (S100β, gray) specificity, respectively. Images are representative of labeling in *n =* 3 animals. Scale bars in **A**–**C** = 50 μm.

**Figure 3:**
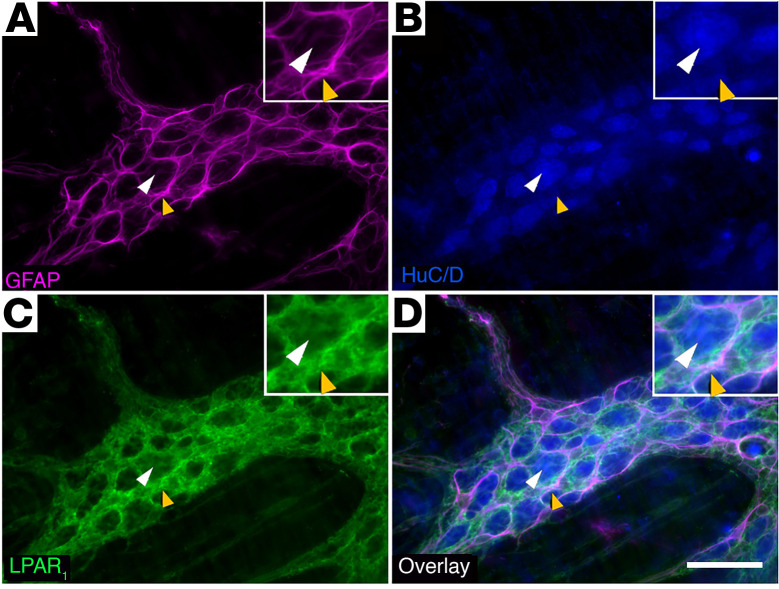
Distribution of LPAR_1_ protein expression in the mouse colon myenteric plexus. Representative example of a myenteric ganglion from the mouse colon labeled with antibodies against glial fibrillary acidic protein (**A**, GFAP, magenta), HuC/D (**B**, blue), and LPAR_1_ (**C**, green). Overlay shown in **D**. Note that LPAR_1_ immunofluorescence is strong in GFAP-positive glia (examples highlighted by yellow arrowheads) and absent in neuron cell bodies (highlighted by white arrowheads). Images are representative of labeling in *n =* 9 animals. Scale bar in **D** = 20 μm and it pertains to **A**–**D**.

We validated the cellular distribution of LPAR_1_ expression in the myenteric plexus of the mouse colon using fluorescence in situ RNA hybridization (RNAscope; Figure 2) and immunofluorescence labeling (Figure 3). In situ hybridization revealed that *Lpar1* probe fluorescence localized to S100β-positive enteric glial cell bodies within the myenteric plexus (Figure 2A). Most, if not all, S100β-positive enteric glia expressed *Lpar1* RNA as indicated by colabeling with the RNAscope probe. In contrast, *Lpar1* RNA was rare or not expressed in peripherin-positive enteric neurons. *Lpar1* probe fluorescence was also observed outside the myenteric plexus, which could indicate *Lpar1* RNA expression by intramuscular glia or the smooth muscle cells themselves. Positive and negative controls for RNAscope probes are shown in Supplemental Figure 2C and labeling with probes targeting *Ret* and *Sox10* is shown in Figure 2, B and C, as a comparison of expression of putative neuronal (*Ret*) and glial (*Sox10*) markers. Immunolabeling for LPAR_1_ protein mirrored the RNA expression profile and strong LPAR_1_ labeling was restricted to GFAP-positive enteric glia and absent in neurons, which express the neuronal marker HuC/D (Figure 3, A–D and Supplemental Figure 2, A and B). Together, these data show that LPAR_1_ protein and RNA are enriched in myenteric glia and are rare or not expressed by most myenteric neurons.

### LPAR_1_ activation stimulates glial Ca^2+^ responses.

**Figure 4:**
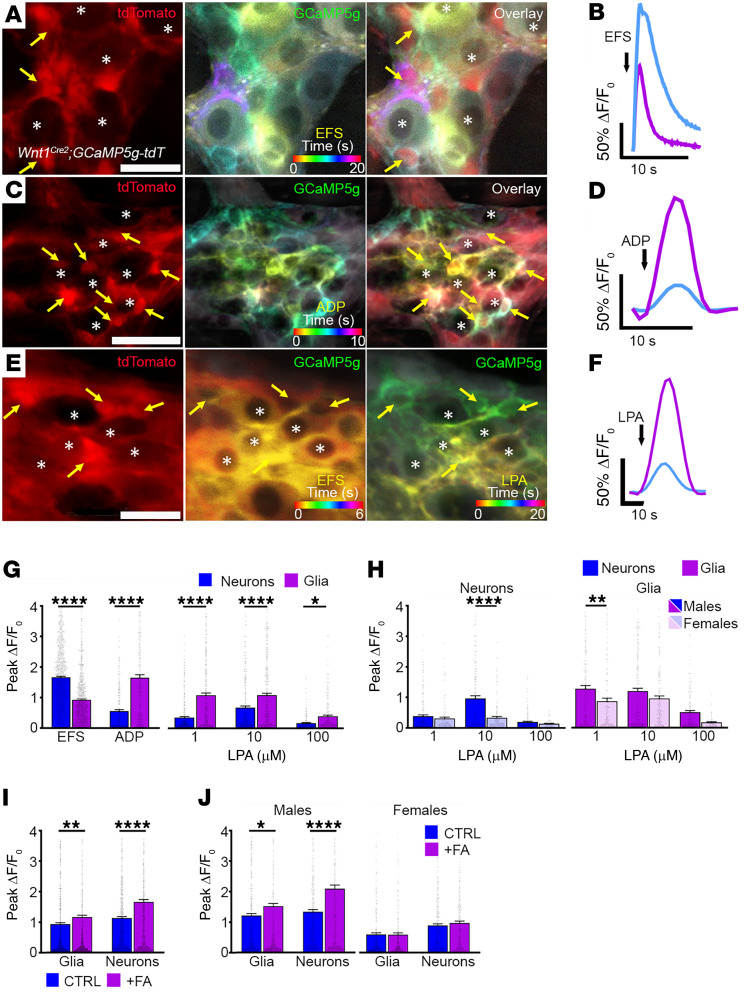
LPAR_1_ activation drives Ca^2+^ responses in myenteric glia. Representative examples of Ca^2+^ responses in single myenteric ganglia from the colons of *Wnt1^Cre2^ GCaMP5g-tdT* mice. (**A**, **C**, and **E**) tdT fluorescence (red, left panels) is high in glia and low in neurons in *Wnt1^Cre2^ GCaMP5g-tdT* mice. GCaMP5g fluorescence (center and right panels) is broadly distributed among neurons and glia. Panels in **A** and **B** (centers) and **E** (center and right) display representative responses (GCaMP5g fluorescence) to stimuli as a temporal color-coded projection. Representative examples of glia (yellow arrows) and neurons (asterisks) that responded to electrical field stimulations (EFS), ADP, or EFS/18:1 LPA are highlighted (**A**, **C**, and **E**, respectively). Note that EFS evokes broad Ca^2+^ activity among enteric neurons followed by activity in enteric glia, while responses to ADP and LPA are predominantly confined to glia. (**B**, **D**, and **F**) Quantification of neuron and glial Ca^2+^ responses evoked by EFS, ADP, and LPA in myenteric ganglia, respectively. (**G**) Summary of EFS, ADP, and LPA-mediated Ca^2+^ responses in myenteric neurons and glia. (**H**) Summary data showing neuronal and glial responses to various concentrations of LPA in samples from male and female mice. (**I**) Summary data showing neuronal and glial responses to 1 μM LPA in control (CTRL) and fluoroacetate-treated (FA) tissues and (**J**) their stratification between male and female mice. *n =* 141–1073 glia and 153–1064 neurons in **A**–**J**; *P < .05, **P < .01, and ****P < .0001, by 2-tailed *t* test and 1-way ANOVA. Scale bars in **A** and **E** = 25 μm and in **C** = 50 μm.

Our findings show conserved genetic expression of *LPAR1/Lpar1* in enteric glia, suggesting that it serves an important role in ENS physiology. Prior work indicated the potential for LPA to evoke Ca^2+^ responses in enteric glia cultured from neonatal guinea pigs and mice (17, 39) but the physiological significance of those in vitro findings remained unclear. Therefore, we tested how activating LPAR_1_ affects activity among neurons and glia in myenteric motor circuits using intact whole mount preparations from *Wnt1^Cre2^ GCaMP5g-tdT* transgenic mice. The *Wnt1^Cre2^ GCaMP5g-tdT* line expresses the Ca^2+^ indicator, GCaMP5g, in enteric neurons and glia, which are easily differentiated based on high tdTomato fluorescence in glia and low or undetectable tdTomato fluorescence in neurons (Figure 4, Supplemental Figure 3, and ref. 20).

We initially stimulated the myenteric plexus with electrical field stimulation (EFS) to elicit broad neuronal activation. Enteric neurons are electrically excitable (Figure 4, A and E) and EFS (70V, 10Hz) provoked a mean Ca^2+^ response measuring ΔF/F_o_=1.67 ± 0.03 (*n =* 1064 neurons) relative to baseline and activated 95% ± 4% of neurons (Figure 4G). These experimental parameters mobilize physiological levels of intracellular Ca^2+^ in neurons, which releases neurotransmitters that activate nearby enteric glia (21). Thus, EFS-mediated neuronal responses were accompanied by a glial response measuring ΔF/F_o_=0.92 ± 0.03 (*n =* 886 glia) and exhibiting similar activation kinetics (Figure 4, A, B, and G). Under these conditions, neurons recruited approximately 82% ± 4% of neighboring glia, highlighting the profound degree to which neuronal responses can influence glial activation states.

To establish a framework for interpreting the effects of LPAR_1_ activation in the intact ENS, we characterized glial Ca^2+^ responses to focally applied adenosine diphosphate (ADP) (Figure 4, C, D, and G). ADP is a potent glial stimulant in the ENS (17, 23, 24, 43) and although *P2ry1* gene expression is observed in both enteric neurons and glia in mice (40, 41), P2Y_1_Rs are either not functionally expressed by neurons or are under tonic inhibition since ADP does not directly activate mouse enteric neurons through P2Y_1_Rs in culture (17). Thus, the effects of ADP on neuron activity likely involve glial activity. ADP evoked glial Ca^2+^ responses measuring ΔF/F_o_ = 1.65 ± 0.099 (*n =* 239 glia; Figure 4, C, D, and G) and recruited 68% ± 10% of glia. ADP-mediated glial responses were accompanied by a delayed phase of neuronal recruitment that was offset by approximately 1 to 2 seconds, measured ΔF/F_o_ = 0.555 ± 0.05 (*n =* 239 neurons), and activated 38% ± 8% of neurons overall. Together, these data show that neurons have the potential to activate glia and vice versa.

After characterizing typical neuronal and glial response patterns to EFS and ADP, we studied the direct effect of 18:1 LPA on the intact ENS (Figure 4, E–G, and I). The 18:1 LPA was selected as it is the most highly produced and longest-lived of the various LPA species generated during inflammation (38), making it an ideal candidate for studying the role of glial LPAR_1_ in the pathogenesis of motility disorders, which may have an underlying inflammatory cause. Application of 1 μM LPA elicited glial Ca^2+^ responses measuring ΔF/F_o_ = 1.084 ± 0.074 (*n =* 333 cells), which was about 3 times greater than the response evoked in neurons (*n =* 312 cells, *P <* 0.0001; Figure 4, F and G). By comparison, 10 μM LPA evoked glial Ca^2+^ responses measuring ΔF/F_o_ = 1.085 ± 0.064 (*n =* 350 cells), which was about 1.6 times greater than the response observed in neurons (*n =* 311 cells; *P <* 0.0001). Despite the apparently greater neuronal Ca^2+^ responses evoked by 10 μM LPA, however, there was no difference in the proportion of neurons recruited at this concentration (36% ± 10% of 311 cells from 9 mice) compared with 1 μM LPA (26% ± 6% of 312 cells from 9 mice; *P =* 0.393). Overall, the immediate LPA-mediated Ca^2+^ response was confined to the glial network (Figure 4, E and F) with neuron activity following, which is consistent with prior cell culture work showing that enteric glia exhibit direct responses to LPA while enteric neurons do not (17). Together, these findings demonstrate that LPA drives glial Ca^2+^ responses in situ and that glial activity evoked by LPA may influence neurons in motor circuits.

We stratified LPA-mediated glial Ca^2+^ responses by sex (Figure 4H) and found that male enteric glia exhibited more robust Ca^2+^ responses following application of 1 μM LPA (ΔF/F_o_ = 1.27 ± 0.103, *n =* 180 cells) compared with females (ΔF/F_o_ = 0.861 ± 0.103, *n =* 153 cells; *P =* 0.0053). While this glial sex effect was not observed at higher concentrations of LPA, neuronal responses to 10 μM LPA were also greater in males (ΔF/F_o_ = 0.962 ± 0.089, *n =* 170 cells) compared with females (ΔF/F_o_ = 0.331 ± 0.043, *n =* 141 cells; *P <* 0.0001). These data suggest sex specificity and concentration-dependent cellular effects of LPAR_1_ in the ENS.

To provide additional evidence that responses to LPA in myenteric ganglia primarily involve glial mechanisms, we exposed samples to fluoroacetate (FA) to test how perturbing glial metabolism affects glial and neuronal responses to LPA. Our previous work shows that acute exposure to FA decreases the number of myenteric glia responding to ADP, but those that still responded exhibit larger peak Ca^2+^ responses, suggesting that this metabolic challenge promotes a reactive-like phenotype (43). We found that FA increased the LPA-mediated peak Ca^2+^ responses in both glia and neurons (Figure 4, I and J). Interestingly, these effects were limited to samples from male mice and were not observed in females, supporting a higher male sensitivity to FA effects (Figure 4J).

### LPAR_1_ modulates gut motility.

Our cellular imaging studies show that LPA acts on glia to affect activity in myenteric circuits that control gut motility. To test whether these cellular responses translate to effects on motility, we modulated LPAR_1_ activation and studied the resulting effects on motility ex vivo.

**Figure 5:**
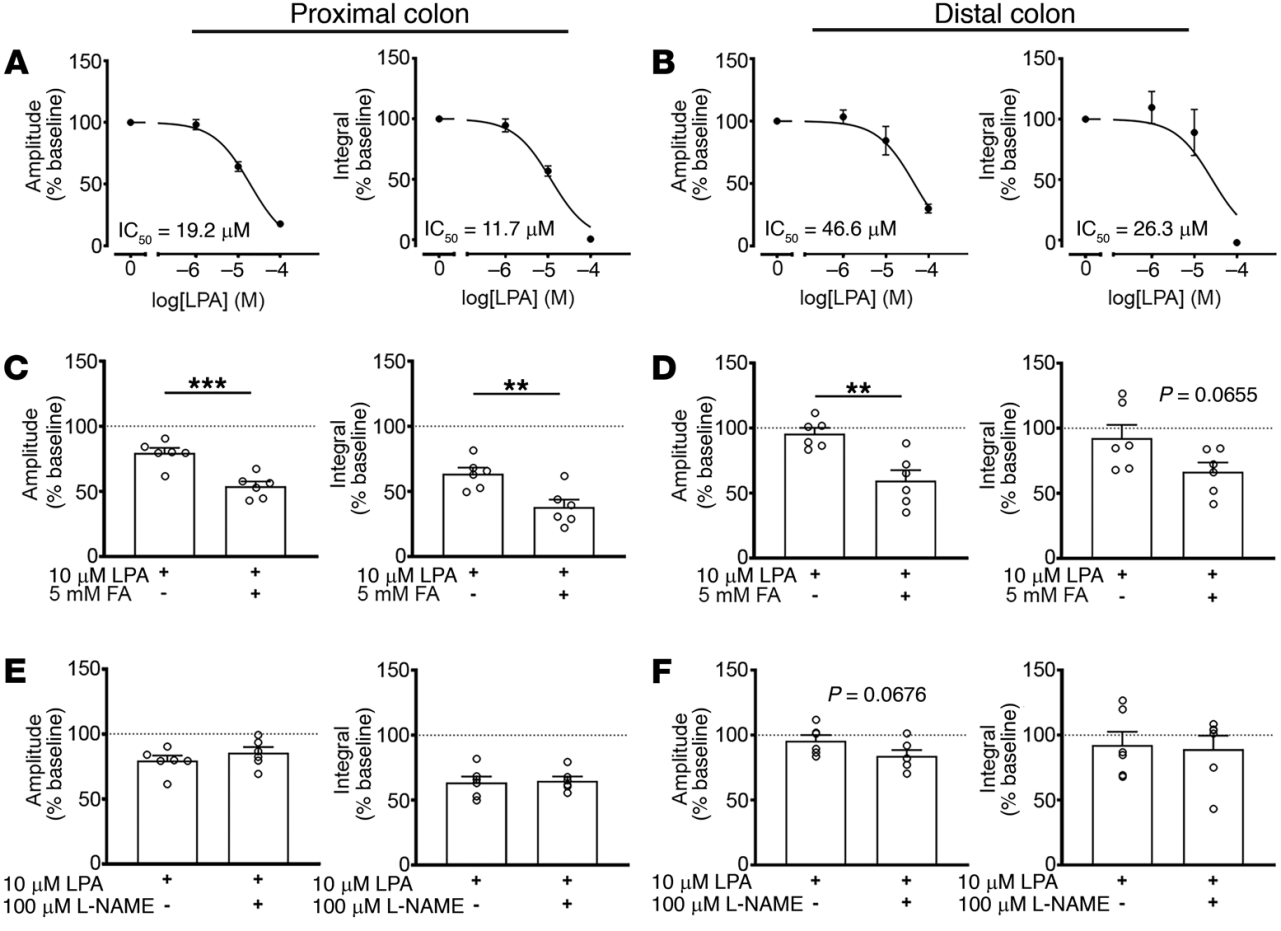
Effects of LPAR_1_-mediated signaling on intestinal motility. (**A** and **B**) Acute stimulation of LPAR_1_ with bath-applied 18:1 LPA attenuates CMC contractile force in a concentration-dependent manner. (**C** and **D**) Impairing glial metabolism with FA exacerbates the inhibitory effect of 18:1 LPA on CMC contractile force. (**E** and **F**) Blocking nNOS activity with L-NAME does not alter the reduction in CMC contractile force following 18:1 LPA. *n =* 5–6 mice in **A**–**F**, **P < .01 and ***P < .001, by 2-tailed *t* test (**C**–**F**).

We assessed lower GI tract motility by measuring the effect of 18:1 LPA on colonic motor complexes (CMCs) (Figure 5, A–F). CMCs are spontaneous interdigestive patterns of colonic contractions that propagate in an oral-to-aboral direction and are dependent on the circuitry of the myenteric plexus (44). Although the exact mechanisms regulating CMC formation remain unclear, measuring CMCs provides insight into the effect of LPA on ENS-dependent GI motility. As such, application of LPA evoked a concentration-dependent reduction in colonic contractility in both the proximal (IC_50_ = 19.2 μM) and distal colon (IC_50_ = 46.6 μM, *n =* 5; Figure 5, A and B and Supplemental Figure 4). As in our cellular imaging studies, this effect was enhanced in the presence of FA (Figure 5, C and D), suggesting an additive effect mediated through glia. Earlier work suggested that some effects of LPA on esophageal motility involve modifications to nitrergic signaling (45). Blocking nitric oxide synthesis with L-NAME had no effect on the LPA-mediated changes in CMCs (Figure 5, E and F). Therefore, it is likely that LPA lessened CMC activity through proinflammatory effects on glia rather than altering neuronal nitrergic signaling.

### Effects of blocking LPAR_1_ in vivo on intestinal motility and ENS pathology.

**Figure 6:**
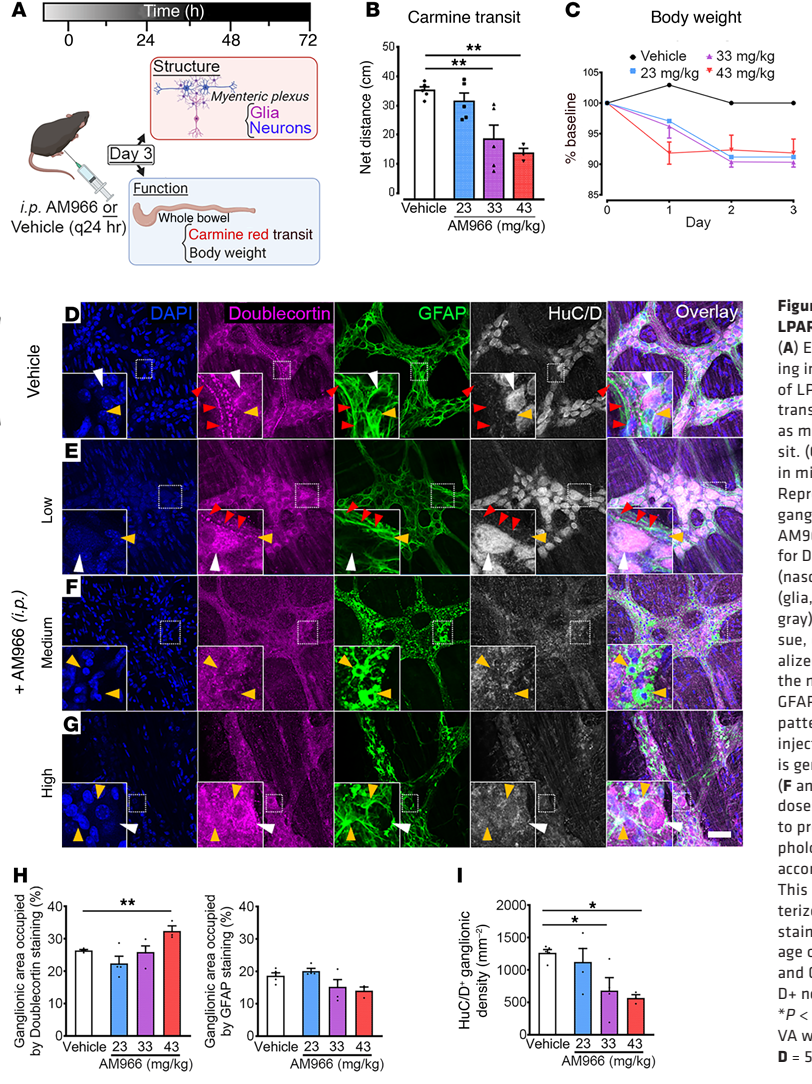
Effects of blocking LPAR_1_-mediated signaling in vivo. (**A**) Experimental paradigm illustrating in vivo pharmacological blockade of LPAR_1_ signaling. (**B**) Whole gut transit in mice treated with AM966 as measured by carmine red transit. (**C**) Body weight measurements in mice treated with AM966. (**D**–**G**) Representative examples of myenteric ganglia from the colons of control and AM966-treated mice immunolabeled for DAPI (nuclei, blue), doublecortin (nascent neurons, magenta), GFAP (glia, green), and HuC/D (neurons, gray). (**D**) In vehicle-treated colonic tissue, doublecortin staining can be visualized in fiber tracts coursing through the myenteric plexus alongside GFAP-positive fibers. This staining pattern is largely conserved in animals injected with low-dose AM966 (**E**) but is generally absent at higher doses (**F** and **G**). Both moderate (**F**) and high doses (**G**) of AM966 injection appeared to promote remodeling of glial morphology in the myenteric plexus with accompanying loss of enteric neurons. This pathological pattern is characterized by diffuse, hyperintense GFAP staining (green-channel). (**H**) Percentage of ganglionic area of doublecortin and GFAP staining. (**I**) Ganglionic HuC/D+ neuron density. *n =* 3–5 mice in **B**–**I**, *P < .05 and **P < .01, by 1-way ANOVA with Dunnett’s test. Scale bar in **D** = 50 μm and it pertains to **A**–**D**.

The in vitro experiments described above likely reflect pathophysiological scenarios when LPA levels rise abruptly (42). To test the contribution of normal low, physiological levels of LPA/LPAR_1_ signaling to gut motility we blocked LPAR_1_ signaling with the selective antagonist AM966 and measured total GI transit time in vivo with carmine red (Figure 6A) (18, 46–48). In vehicle-injected mice, carmine red dye traversed 35.24 ± 1.21cm (*n =* 5) of the total GI tract. Compared with controls, dye transit was drastically reduced in mice following exposure to medium (33 mg/kg: 18.50 ± 4.81cm, *n =* 5; *P =* 0.0014) or high (43 mg/kg: 13.67 ± 1.66cm, *n =* 3; *P =* 0.0014) doses of AM966 (Figure 6B). Together, the in vivo (Figure 6) and in vitro (Figure 5) motility data show that deviations from an optimal physiological level of LPAR_1_ signaling lead to GI dysmotility.

Animals treated with AM966 also exhibited a reduction in total body weight that was consistent between the low- (23 mg/kg), medium- (33 mg/kg) and high-dose (43 mg/kg) treatment groups (Figure 6C). This weight loss followed a consistent time course in all 3 drug treatment groups, plateauing by day 3. Although weight loss could have multiple causes, this observation does agree with the clinical presentation of severe motility disorders such as CIPO where patients often require parenteral nutrition to overcome the inability to have an adequate oral caloric intake and avoid malnourishment. Remarkably, 2 of the 5 mice treated with high-dose AM966 were found dead on day 3 and postmortem necropsies performed in these mice revealed consistent, small bowel distension accompanied by a continuous pattern of transmural hemorrhaging that spared the colon (Supplemental Figure 5A). Furthermore, the fecal pellets recovered in the colons of these mice were large and impacted, indicating that colonic transit was also dysregulated and that the overall effect was greatest in the ileum (Supplemental Figure 5B). This experimental finding is consistent with the overall presentation of small bowel ischemia observed in the surgical setting during functional bowel obstructions, including CIPO. Interestingly, mice treated with low-dose AM966 experienced a similar degree of weight loss as their medium- or high-dose–treated counterparts but did not exhibit reductions in carmine red transit.

To assess whether changes in the ENS contributed to the observed bowel disturbances in AM966-treated mice, we studied architectural changes in the myenteric plexus using immunofluorescence labeling. Compared with vehicle-treated mice, low-dose AM966 did not affect ENS ganglionic expression of GFAP or HuC/D density (Figure 6, D and E). In these cases, GFAP staining exhibited a uniformly reticulated pattern with Hu-positive neurons evenly distributed within this network. Doublecortin staining, which delineates the location of nascent neurons (49), was relatively consistent between these 2 treatment groups and exhibited a linear punctate pattern resembling the varicosities of extrinsic nerve fiber tracts. Importantly, myenteric plexus structure was unaffected by low-dose AM966 as demonstrated by the qualitatively normal GFAP staining pattern.

Compared with vehicle controls, mice injected with medium-dose AM966 also displayed no difference in ganglionic area of GFAP staining. However, this appeared to be accompanied by altered glial cell morphology, which involved process thickening and retraction (Figure 6, D and F). Ganglionic density of HuC/D+ neurons was significantly reduced by approximately 46% (*P <* 0.05; Figure 6I). Given that doublecortin staining was not affected, however, this likely reflects loss of mature enteric neurons without overt effects on the proliferating pool of enteric neurons.

High-dose AM966 exhibited similar ENS alterations characterized by glial process retraction and profound cytoskeletal disarray (Figure 6, G, H, and I). High-dose AM966 increased ganglionic doublecortin staining by 6% (*P <* 0.01) and decreased ganglionic HuC/D+ density by approximately 55% (*P <* 0.05) compared with control. Both medium- and high-dose groups appeared similar to vehicle controls by the absence of systemic inflammation following AM966 injection noted elsewhere (Supplemental Figure 6C). Taken together, these data suggest that in vivo blockade of LPAR_1_ promotes enteric glial dysfunction and loss of mature but not immature enteric neurons within this timeframe of drug exposure.

### Glial LPAR_1_ expression is reduced in human ileum and colon during CIPO.

Our findings in mice show that LPA acts through glial LPAR_1_ to modify ENS activity, that LPA affects CMC activity, and that blocking LPAR_1_ produces a failure of gut motility that involves a loss of normal ENS architecture. While these observations suggest that glial LPAR_1_ plays an important role in GI physiology, its exact relevance to human GI motility disorders remains unclear. We therefore proceeded to investigate whether there is evidence that would support altered glial LPAR_1_ signaling in human motility disorders. Toward this end, we assessed LPAR_1_ expression in the ENS of humans with CIPO, an exemplary severe motility disorder.

**Figure 7:**
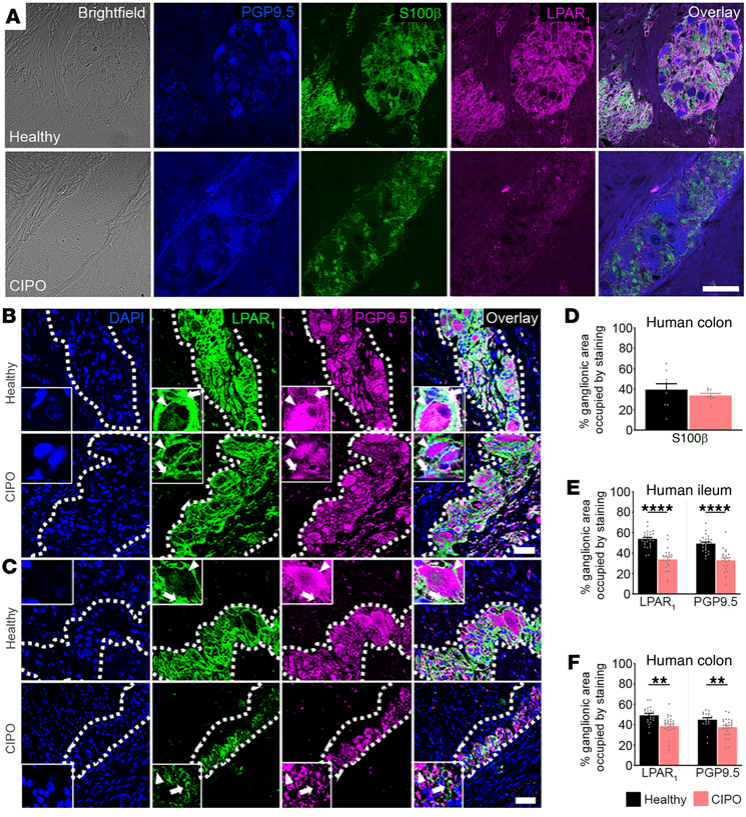
Glial LPAR_1_ expression is reduced in humans with CIPO. (**A**) Representative images of brightfield, PGP9.5 (blue), S100β (green), and LPAR_1_ (magenta) in cross-sections from healthy and CIPO human colons (top and bottom rows, respectively) in 8–9 ganglia from 4 patient samples. Note that LPAR_1_ localizes to enteric glia (S100β) while absent in enteric neurons (PGP9.5). (**B**) Top: Representative images of LPAR_1_ (green) and PGP9.5 (magenta) in the healthy human ileum. LPAR_1_ is expressed by enteric glial cells (white arrow) throughout the myenteric ganglion. By comparison, LPAR_1_ expression is nearly absent from adjacent enteric neurons (white arrowhead), which express high levels of PGP9.5. Bottom: In CIPO, LPAR_1_ and PGP9.5 expression is reduced in glia and neurons, respectively. (**C**) Top: Representative images of LPAR_1_ (green) and PGP9.5 (magenta) expression in the myenteric plexus of the healthy human colon. Like the ileum, LPAR_1_ is localized to regions surrounding neurons, indicating glial-specific expression in the human colon. Bottom: LPAR_1_ and PGP9.5 expression are reduced in CIPO. (**D**) Semiquantification of cross-sectional protein expression of S100β in healthy and CIPO samples of human colons. Semiquantification of cross-sectional protein expression of LPAR_1_ and PGP9.5 in CIPO relative to healthy controls in ileum and colon (**E** and **F**, respectively). *n =* 19–27 ganglia from 4–6 patient samples in **B**–**F**, **P < .01 and ****P < .0001, by 2-tailed *t* test. Scale bar in **A** = 50 μm; scale bar in **C** = 20 μm and it pertains to **B** and **C**.

Full thickness sections of ileum and colon from healthy controls and patients with CIPO (Figure 7, A–C) were colabeled with antibodies against LPAR_1_, S100β to identify enteric glia, and PGP9.5 to identify enteric neurons. LPAR_1_ immunolabeling was robust throughout the myenteric plexus of the human ileum (Figure 7B) and colon (Figure 7, A and C) in samples from healthy controls (*n =* 4). CIPO did not alter S100β expression in the human colon (*P* > 0.5, Figure 7D). In agreement with labeling in mice, LPAR_1_ staining was undetectable in neurons and intense in S100β-positive glia surrounding PGP9.5-positive neuronal cell bodies (Figure 7A), confirming restricted expression of LPAR_1_ protein to enteric glia in humans.

Samples from individuals with CIPO exhibited significantly less LPAR_1_ and PGP9.5 labeling in the ileum (Figure 7, B and E) and colon (Figure 7, C and F) myenteric plexus. Ganglionic area of LPAR_1_ staining in the CIPO myenteric plexus was approximately 20% lower in the ileum (*n =* 4, *P <* 0.0001; Figure 7E) and approximately 11% lower in the colon (*n =* 6, *P <* 0.01; Figure 7F). Measurements of the ENS cross-sectional architecture did not reveal differences in ganglion size or the number of enteric nerve cell bodies between CIPO and healthy tissues (Supplemental Figure 6). Overall, these data confirm that LPAR_1_ is highly expressed by enteric glia in humans and that glial LPAR_1_ signaling may be altered in patients with CIPO.

## Discussion

GI motility disorders such as CIPO are debilitating, common, and poorly understood conditions. Here, we demonstrate that LPA signaling through LPAR_1_ may contribute to GI motility disorders and, in particular, CIPO. LPAR_1_ expression is concentrated in enteric glia within the ENS motor control centers of mice and humans. In mice, activating LPAR_1_ drives Ca^2+^ responses in enteric glia and modifies ENS-driven motor functions such as the CMC. Blocking LPAR_1_ in mice promotes GI dysfunction that is reflected by decreased bowel transit and ENS structural alterations that indicate neuropathy and gliopathy. In humans, glial LPAR_1_ protein expression is reduced in CIPO, suggesting a possible contribution to severe motility disorders. Together, these findings support a novel role for enteric glial LPAR_1_ in the regulation of GI motility and the pathogenesis of CIPO.

LPAR_1_ is pleiotropically expressed (42) and whole-body knockout studies suggest that LPAR_1_ regulates tight junction protein expression in the colon (50). While the motility-related effects of LPAR_1_ could therefore have been due to barrier disruptions, it is unlikely that this is the case for several reasons. Firstly, enteric glia have the potential to modulate epithelial barrier function (51, 52) such that compromised barrier integrity and susceptibility to DSS-induced colitis in whole-body *Lpar1* knockout mice (50) could be amenable to dysregulated enteric glial LPAR_1_ signaling, per se. In support, similar effects on barrier function and susceptibility to DSS-induced colitis are observed in animals lacking *Entpd2* (NTPDase2), which is primarily expressed by enteric glia (52). In addition, our in vivo experiments showed that AM966 triggers fulminant jejunoileitis that spares the colon. Given the putative role of colonic LPAR_1_ in maintaining the epithelial barrier, it was surprising that we did not observe any gross colonic abnormalities in AM966-treated mice. Last, whole-body *Lpar1* knockout animals exhibit impaired feeding behaviors (53) and wound-healing responses (37), raising the possibility that the GI phenotype observed was due to extrinsic factors.

Although LPAR_1_ blockade in mice caused functional obstruction, stimulating LPAR_1_ attenuated CMCs but also provoked Ca^2+^ responses in glia. These differences likely reflect the physiological versus pathophysiological functions of LPA/LPAR_1_ signaling that are dose dependent. The in vivo data showing that LPAR_1_ blockade causes intestinal dysfunction shows that some level of LPAR_1_ signaling is necessary to maintain normal gut functions and the integrity of the ENS. LPA levels are low in this physiological setting but increase substantially during inflammation (42). The in vitro experiments where glia or intestinal preparations were stimulated with LPA are a better representation of a pathophysiological insult when LPA levels rise abruptly. For example, stimulating astrocytes with similar concentrations of LPA promotes proinflammatory responses downstream of strong Ca^2+^ responses induced by LPAR_1_ receptors (54). LPA also induces production of proinflammatory mediators such as COX-2 and IL-8 from human colon cancer cell lines (37) and the main acute effect of these and other proinflammatory mediators is to decrease colonic motility (55, 56). The link between acute LPA surges and proinflammatory glial responses is strengthened by our results showing additive effects of LPA and FA. Enteric glia respond to the acute metabolic challenge induced by FA through protective responses indicative of a reactive-like phenotype (43). The data show that FA exacerbates the effects of LPA on glial Ca^2+^ responses and on motor function, which is consistent with additive effects through proinflammatory glial mechanisms. These data also support the idea that acute LPA exposure at the concentrations used here models mechanisms that occur during a pathophysiological insult while blocking LPAR_1_ reflects its physiological roles in vivo.

It is also possible that the differences between the effects of LPAR_1_ stimulation and blockade can be reconciled by the fact that LPAR_1_ activation drives internalization of the receptor into early endosomes (57, 58). Physiologically, this likely serves to dampen receptor activity when local agonist concentrations are exceedingly high, as is the case during acute inflammation. Moreover, LPAR_1_ endosomes activate noncanonical signaling programs that promote long-term changes in structural gene expression (57). Thus, in the presence of high LPA concentration, the ensuing receptor internalization may shift the second messenger cascade away from G_q/11_ and toward G_13_, which in turn can initiate changes in cell morphology (59). Alternatively, it is also possible that glial LPAR_1_ affects enteric neuronal subtypes involved in regulation of smooth muscle tone. In the feline lower esophageal sphincter, for instance, LPA potentiates sphincter relaxation by blocking presynaptic uptake of nitric oxide (45). However, it is unlikely that the reduction in colonic motility that we observed was due to a similar effect on nitrergic neurons since blocking NO production with L-NAME did not affect LPA-mediated CMC changes.

Our data provide compelling evidence that LPAR_1_ is robustly expressed by enteric glia and may contribute to motility disturbances in mice and humans. The mechanism through which this occurs is unclear, but may be dictated by the intensity and duration of the stimulus. In the CNS and periphery, neuroglial LPAR_1_ has developmental and pathological roles involving control of neurocircuit architecture. For instance, LPAR_1_ guides Schwann cell myelination of motor neurons (60) while satellite glial LPAR_1_ initiates neuropathic pain by promoting cytoskeletal retraction (32). Thus, enteric glial LPAR_1_ activity may serve a similar role in maintaining the structural integrity of the ENS; a possibility that is supported by the profound decay in ENS architecture seen in AM966-injected mice. One corollary of this is that glial LPAR_1_ is constitutively active in maintaining ENS architecture, though we suspect that basal levels of interstitial LPA are low and tightly regulated.

Many disorders of gut-brain interactions feature a remote history of acute infectious enteritis (61) and biolipids, including 18:1 LPA, are robustly generated during the acute phase of inflammation. Activation of enteric glial LPAR_1_ may serve as the bridge linking early tissue damage to long-term remodeling of ENS architecture. This would be consistent with the function of glial LPAR_1_ in shaping the neural landscape during development, but also with its purported role in resculpting peripheral neurocircuitry during disease. In accordance with this, the reductions in *Lpar1* and *Enpp2* gene expression observed following acute colitis may constitute an early perturbation that, if sustained, lead to the histopathological changes seen in patients with CIPO. Indeed, tissue levels of 18:1 LPA remain uniquely elevated in mice following partial ligation of the sciatic nerve weeks following the initial injury (38). It is therefore conceivable that persistence of LPA in the ENS could potentiate the initial tissue damage. If confirmed, this would imply that the severity of disorders of gut-brain interactions is governed by the degree and persistence of glial LPAR_1_ dysregulation and that CIPO simply lies on the extreme end of this spectrum. Whether glial LPAR_1_ is dysregulated in the context of other GI dysmotilities, however, is an important next step that should be further investigated.

The finding that enteric glial LPAR_1_ expression is conserved across different species suggests that this receptor fulfills a critical role in GI physiology. Considering our clinical and functional data, this role likely involves modulation of gut motility possibly through long-term regulation of ENS architecture and acute modulation of Ca^2+^ handling dynamics. Ultimately, our study identifies a novel role for LPA and enteric glia in the pathogenesis of GI dysmotility and unveils a new target that may be of diagnostic and therapeutic benefit in the clinical management of patients with CIPO.

## Methods

### Human sample collection.

Full thickness colon (*n =* 6) or ileum (*n =* 5) (one patient had both ileum and colon) tissue was obtained from adults (*n =* 10; 8 females, 2 males; age range: 22–73 years) with a diagnosis of CIPO established via clinical assessment, radiological tests (mainly abdominal CT scan) and small bowel manometry at St. Orsola-Malpighi Hospital from 2014 to 2019.

Comparable tissue samples (*n =* 4 ileum; *n =* 4 colon), obtained from 4 patients (2 females, 2 males; age range: 48–73 years) (asymptomatic for previous GI symptoms and referred to surgery for noncomplicated GI tumors) were used as controls. All tissue specimens were immediately fixed in cold neutral 4% formaldehyde (Kaltek), paraffin- embedded, cut into 5-μm-thick sections, and mounted serially on poly-l-lysine–coated slides (Thermo Fisher Scientific).

### Clinical features of patients.

Patients with CIPO secondary to infectious, neurological, metabolic, systemic autoimmune, and paraneoplastic conditions as well as cases secondary to known genetic abnormalities were excluded. Thus, all patients included in this study were idiopathic in origin. The clinical diagnosis of CIPO was established on the basis of a chronic (>3 months), severe symptom complex mimicking mechanical small bowel obstruction, and (at least on one occasion) radiological evidence of air-fluid levels or dilated small bowel loops.

Prior to laparoscopic surgery for tissue collection, patients completed a clinical questionnaire reporting the following data, symptoms, and signs: age, sex, BMI, onset of symptoms, number of subobstructive episodes that occurred prior to surgery, abdominal distension, abdominal pain, nausea, vomiting, fullness, early satiety, constipation, diarrhea, esophageal involvement (i.e., motor abnormalities assessed by standard esophageal manometry), gastroparesis (established by scintigraphic gastric emptying), small intestine bacterial overgrowth (SIBO) (determined by glucose/lactulose breath test), urinary symptoms, and small bowel dilation (detectable at abdominal CT scan or MRI). Medications used by each subject were also recorded. These features are summarized in Table 1 and Table 2.

In 4 patients with CIPO, the ileum was the main site of the disease with at least 3 of 4 patients reporting symptoms of nausea, vomiting, abdominal fullness, early satiety, diarrhea, or constipation. Of these ileum biopsies, only 1 derived from a male patient while the remaining 3 were from female patients. In a separate group of 6 patients with CIPO, the disease was mainly localized to the colon. All patients with colonic CIPO experienced constipation, while diarrhea was reported by only 1 patient. In addition, 4 patients with colonic CIPO reported experiencing mild-to-moderate grade abdominal pain while 1 patient had severe abdominal pain. Importantly, the BMI of patients with CIPO affecting predominantly the ileum (16.28 ± 1.18, *n =* 4) was lower than that of patients where CIPO affected mainly the colon (20.40 ± 0.39, *n =* 6; *P =* 0.033) suggesting differences in long-term nutritional status between the 2 groups.

### Animal use.

Transgenic *Wnt1^Cre^ GCaMP5g-tdT* mice were generated by breeding *Wnt1Cre2* mice (B6.Cg-*E2f1^Tg(Wnt1-cre)2Sor^/J*; The Jackson Laboratory; RRID:IMSR_JAX:022501) with *GCaMP5g-tdT* mice (B6;129S6-*Polr2a^Tn(pb-CAG-GCaMP5g,-tdTomato)Tvrd^/J*; The Jackson Laboratory; RRID:IMSR_JAX:024477). Male and female animals were used between 10 and 14 weeks of age and were housed in a temperature-regulated facility on a 12 hour light/dark cycle and provided ad libitum access to standard chow and water. Mice were genotyped by Transnetyx Inc. WT C57/Bl6 mice were used for immunohistochemistry and motility-related experiments.

### Mouse colonic tissue isolation and processing.

Live colon tissue was collected from euthanized mice in cold Dulbecco’s modified Eagle medium (DMEM) before being further processed for use in Ca^2+^ imaging, immunohistochemistry, or CMC studies.

Isolated colonic tissue was longitudinally slit open along the mesenteric border and pinned out using insect pins in a 35 mm dish coated with Sylgard. The tissue was then fixed with Zamboni’s fixative overnight at 4°C for subsequent immunohistochemical studies or with 4% paraformaldehyde (4% PFA) for RNAscope. For Ca^2+^ imaging studies, the epithelium was removed and whole-mount circular muscle-myenteric plexus (CMMP) preparations were generated by flipping the tissue and removing the longitudinal muscle layer by microdissection as described in prior work (20).

### Immunohistochemistry.

Human tissues were deparaffinized with xylene and rehydrated in a graded ethanol series. Antigen retrieval was performed using sodium citrate buffer solution (10 mM sodium citrate, 0.05% Tween-20, pH 6.0), which was preheated on a hotplate to a constant temperature of 95°C to 100°C.

Following fixation, mouse colon was micro-dissected as described in prior work (24). In brief, the epithelium was removed along with the circular smooth muscle layer, thereby generating a longitudinal muscle-myenteric plexus (LMMP) preparation. For both human and fixed mouse tissues, samples were incubated for 40 minutes with a blocking solution containing 4% normal goat serum, 0.4% Triton X-100, and 1% bovine serum albumin dissolved in PBS. Tissues were then incubated overnight at 4°C with the following antibodies: chicken anti-GFAP (1:1000; catalog ab4674, Abcam), rabbit anti-LPAR1 (1:250; catalog ab23698, Abcam; ref. 3), guinea pig anti-PGP9.5 (1:500; catalog GP14104, Neuromics), biotinylated anti-HuC/D (1:200; catalog A21272, Invitrogen), mouse anti-peripherin (1:100; catalog sc-377093, Santa Cruz Biotechnology), rabbit anti-S100β (1:200; catalog ab52641, Abcam), or rabbit anti-doublecortin (1:200; catalog ab18723). On the day of imaging, samples were incubated with the following fluorescently conjugated secondary antibodies for 2 hours: Alexa 488 donkey anti-chicken (catalog 703-545-155, The Jackson Laboratory), Alexa 488 donkey anti-rabbit (catalog 711-545-152; The Jackson Laboratory), Alexa 594 donkey anti-rabbit (catalog 711-585-152; The Jackson Laboratory), Alexa 594 donkey anti-guinea pig (catalog 706-585-148; The Jackson Laboratory), Alexa 594 goat anti-chicken (catalog 103-585-155; The Jackson Laboratory), streptavidin-Alexa 594 (catalog 016-580-084; The Jackson Laboratory), streptavidin-Alexa 488 (catalog 016-540-084; The Jackson Laboratory), Alexa 647 donkey anti-rabbit (catalog 711-605-152; The Jackson Laboratory), donkey anti-chicken Cy5 (catalog 703-175-155; The Jackson Laboratory), and/or streptavidin Dylight Alexa 405 (catalog 016-470-084; The Jackson Laboratory). All secondary antibodies were used at a dilution of 1:400. Prior to imaging, tissues were mounted in 4′,6-diamidino-2-phenylindole (DAPI) fluoromount G (catalog 0100-20; Southern Biotech).

The specificity of rabbit anti-LPAR1 antibody has been confirmed elsewhere (62) but was further demonstrated here by conducting a preadsorption assay between the primary antibody and its immunizing peptide sequence (catalog 10006984, Cayman) according to manufacturer instructions. Briefly, this entailed incubating the anti-LPAR1 antibody with the immunizing peptide (GGYLPFRDPNSEENSNDIAL) in a 1:1 ratio (vol/vol) for 1 hour at room temperature with occasional mixing. The antibody-peptide cocktail was then diluted to the usual 1:250 antibody dilution and the remaining protocol proceeded as previously described.

### Fluorescence in situ hybridization (RNAscope).

Colonic LMMP tissue for RNAscope was generated following the fixation and dissection procedures described above (see sections “Mouse colonic tissue isolation and processing” and “Immunohistochemistry”). RNAscope was performed using the Advanced Cell Diagnostics (ACD) RNAscope 2.5 HD Assay—RED (catalog 322350) according to the manufacturer’s instructions with adjustments for colonic LMMP tissue. Briefly, tissue was dehydrated and subsequently rehydrated by a serial ethanol gradient (25%, 50%, 75%, 100% in PBST) before H_2_O_2_ treatment. Tissue was then digested with Protease III for 45 minutes and incubated with probes for *Lpar1* (ACD, catalog 318591), DapB (ACD, catalog 310043), Ppib (ACD, catalog 313911), Ret (ACD, catalog 431791), or Sox10 (ACD, catalog 435931) overnight at 40°C. All RNAscope steps were performed in a 96-well plate while wash steps were performed in a 48-well plate for 3 × 5 minutes. Following completion of the RNAscope protocol, immunohistochemistry and tissue mounting were performed as described above.

### Ca^2+^ imaging.

Ca^2+^ imaging studies were conducted in CMMP preparations, which were perfused with Kreb’s buffer (37°C) at a constant rate (2–3 mL/min). Unless otherwise specified, all imaging studies were conducted on an upright Olympus BX51WI fixed-stage microscope. Ganglia were viewed at 20× through a wide-field water-immersion objective lens (Olympus XLUMPLFLN20xW, 1.0 numerical aperture). Illumination for fluorescence imaging was provided by a DG4 Xenon light source (Sutter Instrument).

GCaMP5g photoexcitation was filtered through a 485/20 nm band-pass filter, and emitted light was filtered through a 515 nm long-pass filter. tdT signal was excited by light filtered through a 535/20 nm band-pass filter and reflected tdT signals were filtered through a 610/75 nm band-pass emission filter. Imaging data were acquired at a frame rate of 0.2 frame per second using a Neo sCMOS camera (Andor). In some cases, confocal video fluorimetry was performed using a confocal microscope (Nikon A1R HD25; Nikon; Supplemental Figure 4), which were imaged through a 20× Nikon objective lens (CFI Apochromat LWD Lambda20xC WI, 0.95 numerical aperture). Confocal images were captured using a Nikon DS-Ri2 digital camera (Nikon) and recorded using NIS-Elements C software (Nikon). For epifluorescence and confocal fluorescence imaging studies, all data were saved on a personal computer running Windows 10 (Microsoft Corporation) and MetaMorph (Molecular Devices) and exported as .tiff stacks for analysis with Fiji software (NIH). To inhibit glial metabolism, some CMMP whole mounts were incubated with FA (5 mM dissolved in DMEM; 37°C; 5% CO_2_, 95% air) for 2 hours before tissue imaging as previously described (43). 

### Electrical field stimulation.

Electrical field stimulation (EFS) was used to activate neurons using electricity. This was accomplished by applying depolarizing pulses to the tissue using 2 platinum wires using a GRASS S9E Electrical Stimulator with the following parameters: +70V, 10Hz, and 0.1 ms duration.

### Local drug application.

ADP or 18:1 LPA were applied locally to the ganglion surface to provoke agonist responses. To do this, glass capillary applicators were fabricated with a pipette puller (P-87 Flaming-Brown Micropipette Puller, Sutter Instruments Corporation) and back-filled with drug dissolved in Kreb’s buffer. Drugs were then applied using very gentle positive pressure that was applied with a 1-mL syringe connected to a pipette holder. This approach delivered picoliter volumes of drug and only affected the ganglion within the field of view. We confirmed that the shear fluid stress associated with drug application did not activate neurons or glia under these conditions (not shown).

### Solutions and chemicals.

DMEM was obtained from Life Technologies. Unless otherwise noted, all reagents were obtained from Sigma. The composition of Kreb’s solution used during imaging experiments was as follows: 121 mM Na^+^, 4.9 mM K^+^, 25 mM NaCO_3_^–^, 1.2 mM Mg^2+^, 2.5 mM CaCl_2_, 1.2 mM NaHPO_3_^–^, 11 mM d-glucose, and 1 mM pyruvate. Kreb’s solution was titrated to pH 7.4 with NaOH. 1-oleoyl-2-hydroxy-sn-glycero-3-phosphate (18:1 LPA; Avanti Polar Lipids, Inc.) was dissolved in 50% ethanol (vol/vol) according to the manufacturer’s instructions, and care was taken to avoid surpassing its critical micelle concentration. AM966 (Cayman Chemical Company; catalog 22048) was dissolved in DMSO to a final stock concentration of 50 mM. Glial metabolism was inhibited with the drug sodium fluoroacetate (FA, 5 mM dissolved in DMEM) for 2 hours and 1 hour in Ca^2+^ imaging and colonic motor complexes, respectively. Nitric oxide synthase (NOS) was inhibited by l-NAME (Nω-nitro-l-arginine methyl ester, Cayman) at a final concentration of 100 μM in DMEM.

### AM966 injections and carmine transit studies.

Age-matched males and females were randomly assigned to 1 of 4 possible treatment groups: low-dose AM966 (22 mg/kg), medium-dose AM966 (33 mg/kg), high-dose AM966 (43 mg/kg), or vehicle. WT C57/Bl6 mice were then injected intraperitoneally with the LPAR_1_ antagonist AM966 (catalog 22048; Cayman) every 24 hours for 3 days, or vehicle. The vehicle control consisted of DMSO dissolved in sunflower oil.

After 3 days of continuous exposure to AM966, the in vivo effect of sustained, subchronic LPAR_1_ blockade was assessed by determining the whole-bowel transit of carmine red dye. As described elsewhere (18, 47, 48), mice were administered 200 μL of 6% carmine red solution in water supplemented with 0.5% methylcellulose by oral gavage. After 1 hour, mice were euthanized and the entire GI tract was carefully but rapidly removed and laid out at neutral length on an all-white surface. The carmine red wavefront was then grossly visualized within the gut lumen as a discrete, bright-red bolus that was immediately trailed by a dye-free bowel segment, and its distance relative to the gastric fundus was measured. Additional parameters that were noted included total bowel length, splenic weight, location of the ileocecal pouch, and the presence of strictures or serosal hemorrhages. The colonic regions of these tissues were then collected in drug-free DMEM for subsequent fixation and further immunohistochemical characterization of ENS architecture.

### Colonic motor complexes.

Colonic motility was assessed in response to LPAR1 receptor activation ex vivo. Briefly, whole intact colon was rapidly collected in drug-free DMEM/F-12 maintained at 37°C. The oral and aboral ends of the colon were then mounted in place on a stainless steel holding rod after lumen contents were flushed. Two force transducers (Grass Instruments) were connected to the gut wall via surgical silk approximately 2 cm apart. The baseline tension was calibrated to 0.5 g and spontaneous CMC production was monitored for a period of 20 minutes before experiments were conducted. The last 5 minutes of this acclimatization period was used as a baseline for analysis, which was conducted in LabChart 8 (ADInstruments). Drugs were added cumulatively to the bath and the resultant changes in CMC amplitude and CMC integral were measured in relation to baseline.

### Statistics.

All analyses were conducted in Fiji. Briefly, video analyses were conducted after background-subtracting recordings, which were first stabilized using the StackReg plugin (http://bigwww.epfl.ch/thevenaz/stackreg/). The tdT channel imaged at each experiment’s outset served to identify the location of glial cells and was used to generate corresponding regions of interest (ROIs). Neuronal ROIs were identified by exclusion of glial ROIs, and manually selected based on morphological features. All glial and neuronal ROIs were saved. Ca^2+^ responses were measured using these ROIs and exported to Microsoft Excel (Microsoft Corporation). Unless otherwise indicated, values are reported as fold-change in mean cellular fluorescence intensity relative to baseline florescence intensity (ΔF/F_o_) ± SEM. For Ca^2+^ imaging studies, sample size denotes the number of cells responding under those experimental conditions with the number of mice used is included where appropriate. For all experiments, approximately 30 to 40 neurons and a comparable number of glia were studied per ganglion, with at least 1 to 2 ganglia used per mouse under each recording condition. The total number of glial cells or neurons are represented by *n* values.

Cell counts and ganglionic expression data were analyzed offline using ImageJ software. Cell counts were performed using the cell counter plug-in of ImageJ software. Enteric neuron numbers are presented as ganglionic packing density, which was calculated by tracing the ganglionic area and counting the number of HuC/D-immunoreactive neurons within the defined ganglionic area. Relative ganglionic protein expression was measured by recording the mean gray values of Doublecortin and GFAP fluorescence within a defined ganglionic area. Fluorescence density (integrated density) was calculated as the product of area and mean gray value and is reported as the intensity in arbitrary fluorescence units per squared micrometer of the ganglionic area. Cell counts and ganglionic expression data were performed on a minimum of 10 ganglia per animal and averaged to obtain a value for that animal. The number of animals in each experiment is represented by *n* values.

Data were analyzed using GraphPad Prism 9 (GraphPad Software Inc.) and are shown as mean ± SE or mean with min/max for floating bars. Statistical testing involved 1-way ANOVA with Dunnett’s multiple comparison test or unpaired 2-tailed Student’s *t* tests. *P* less than 0.05 was considered significant. Welch’s correction was applied as needed when data variability was unequal between groups being compared. Grubb’s test was used to compute statistical outliers, which were excluded from the final analysis only if the confidence threshold of α = 0.05 was surpassed. Custom illustrations were designed in BioRender software (https://biorender.com/).

### Study approval.

All experimental procedures on human samples were approved by the Ethics Committee of St. Orsola-Malpighi Hospital for handling and analysis of tissue samples from patients with severe gut dysmotility (EM/146/2014/O). All animal experiments were conducted according to guidelines established by the NIH *Guide for the Care and Use of Animals* (National Academies Press, 2011). All protocols were approved by the Michigan State University IACUC.

## Author contributions

FG, RDG, and BDG conceived and designed research. MMA, JLM, CD, and LS performed experiments. MMA, JLM, CD, LS, FG, RDG, and BDG analyzed data, interpreted results of experiments, edited and revised the manuscript, and approved the final version of manuscript. MMA, JLM, and CD prepared the figures. MMA and JLM drafted the manuscript.

## Supplementary Material

Supplemental data

## Figures and Tables

**Table 2:**
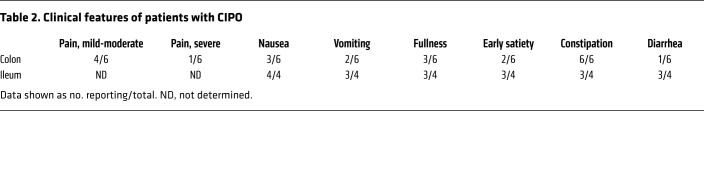
Clinical features of patients with CIPO

**Table 1:**
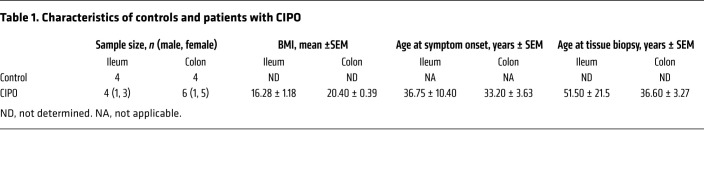
Characteristics of controls and patients with CIPO

## Version 1 02/15/2022

Electronic publication
