# Supplementary material for: LPAR1 regulates enteric nervous system function through glial signaling and contributes to chronic intestinal pseudo-obstruction
Source: J Clin Invest. 2022 Feb 15;132(4):e149464. doi: 10.1172/JCI149464 (PMC8843750; doi:10.1172/JCI149464)
Supplement: Supplemental data [file jci-132-149464-s117.pdf]

Supplementary Figures & Legends

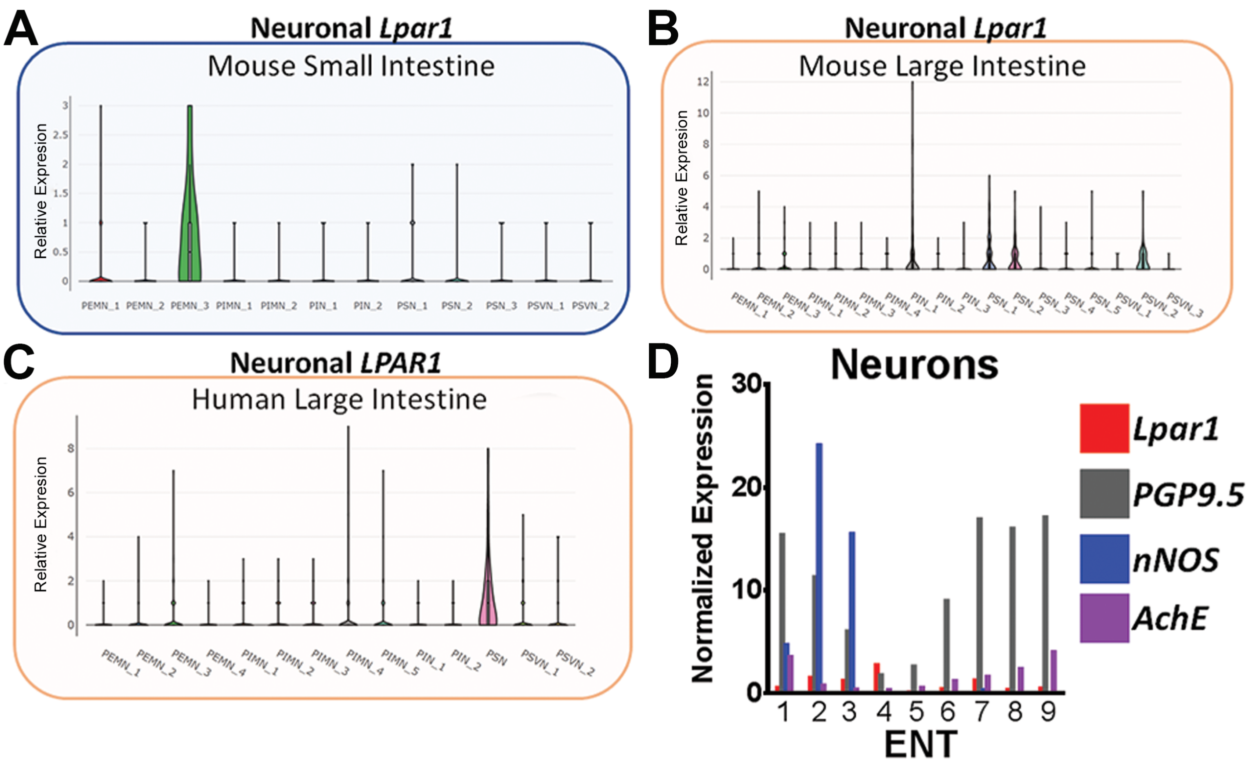

Supplementary Figure 1. ***Lpar1/LPAR1*** **gene expression in enteric neurons.** (**A**) Murine enteric neurons express very low or undetectable levels of *Lpar1*, except for one subtype (*left panel*, green violin plot). This pattern of low neuronal *Lpar1* expression is evident in both the small and large intestine. (**B**) Similarly, *LPAR1* gene expression is low or not detected within enteric neurons of the human large intestine. Data are compiled from a single-cell RNA-sequencing database generated by ref #41. Violin plots were generated using the online single cell database located at (https://singlecell.broadinstitute.org/single_cell). (**c**) Enteric neuronal *Lpar1* expression in the mouse small intestine is far surpassed by classical neuronal markers (*Pgp9.5*, *nNOS*, *AchE*). Data were compiled from the single cell transcriptional profile generated by ref #40, available at http://mousebrain.org/genesearch.html.

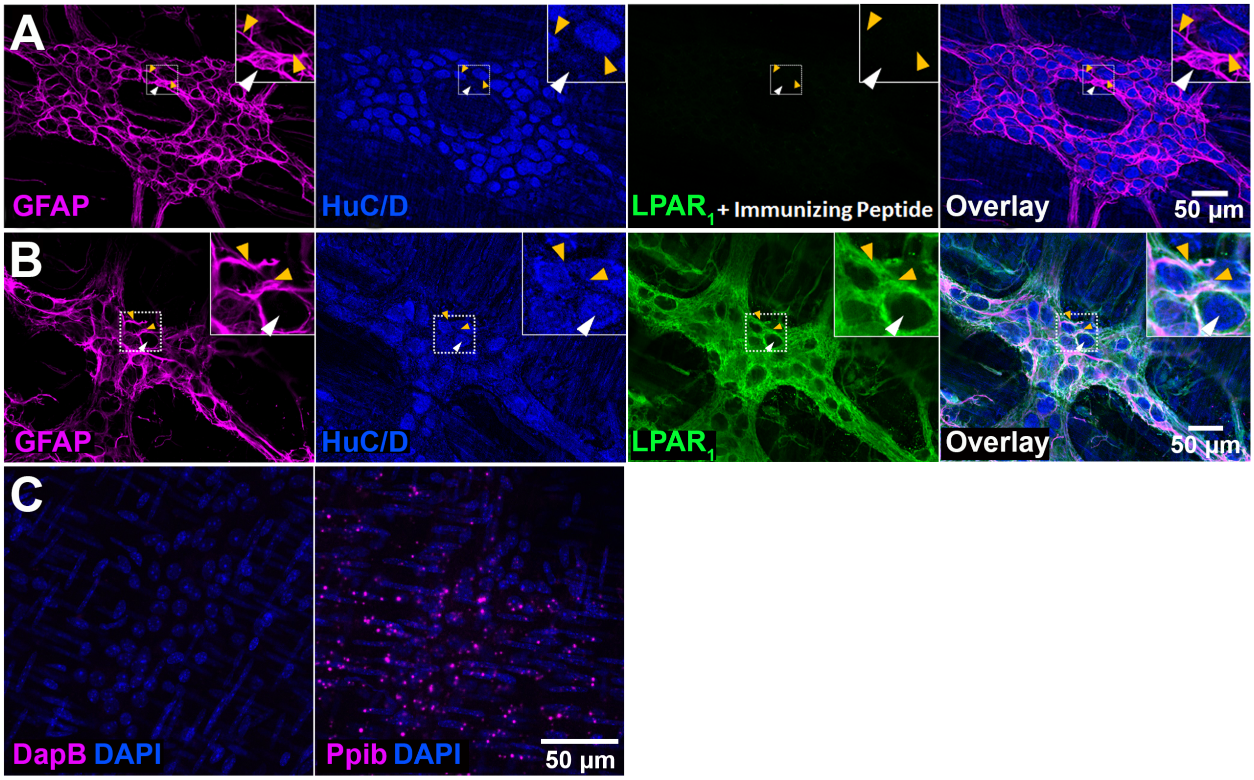

<u>Supplementary Figure 2</u>. Specificity of $LPAR_1$ antibody. (**A**) $LPAR_1$ staining is entirely abrogated when the probing antibody is blocked with the immunizing peptide, suggesting specificity in the staining pattern. (**B**) A representative example of a murine myenteric ganglion triple-stained for GFAP (magenta), HuC/D (blue) and $LPAR_1$ (green). Note the majority of $LPAR_1$ is expressed on the surface of enteric glia delineated by their GFAP+ cytoskeletons (yellow arrows) and not in HuC/D+ neurons (white arrows). (**C**) Negative (left, *DapB*) and positive (right, *Ppib*) RNAscope controls for murine tissue. Images are representative of labeling in a minimum of n = 3 mice.

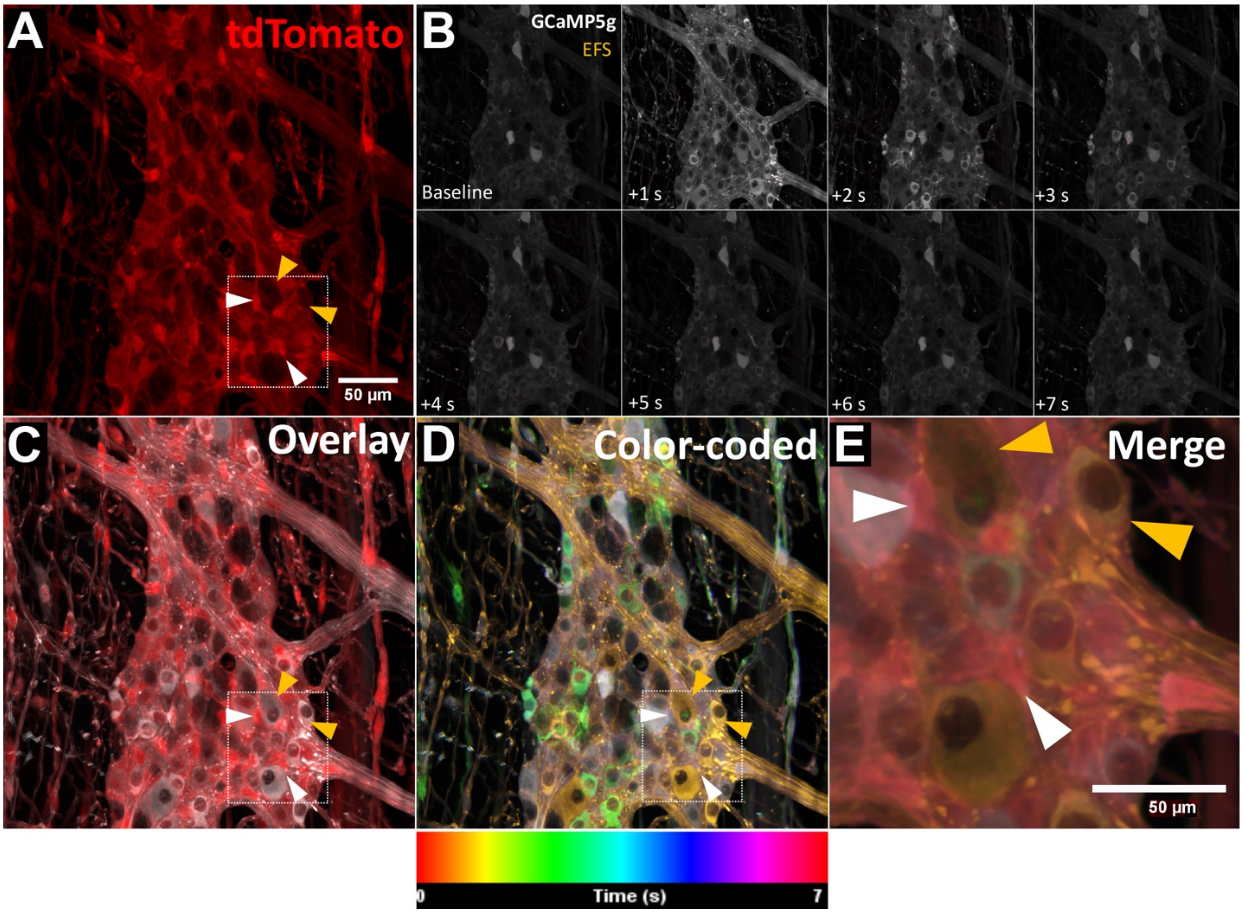

__Supplementary Figure 3__. **tdTomato reporter expression is predominantly localized to enteric glia in *Wnt1^{Cre};GCaMP5g-tdT* mice while GCaMP5g expression is comparable between neurons and glia**. (**A**) Expression of tdTomato in the myenteric plexus is highest in enteric glia. Enteric glia are differentiated from neurons based on their distinct morphologies. Glia exhibit small, oblong cell bodies with little cytoplasm that surround the much larger cell bodies of enteric neurons which have large circular nuclei, and more cytoplasm. The spatial difference in tdTomato expression helps to further distinguish enteric glia from neurons, which are electrically excitable (see **B-E**). (**B**) 7-second montage depicting Ca^{2+} responses following electrical field stimulation (EFS). Much of the resultant Ca^{2+} signal is localized to regions where tdTomato expression is low or absent. (**C**) Maximum intensity projection of the Ca^{2+} response evoked by EFS, overlayed with regional tdTomato expression in that ganglion. (**D**) Color-coded temporal projection of the Ca^{2+} response provoked by EFS. (**E**) White arrowheads depict tdTomato-high

regions corresponding to enteric glia, which do not directly respond to EFS. Orange arrowheads depict locations of enteric neurons that are robustly activated by EFS. Images are representative of a minimum of n = 3 mice.

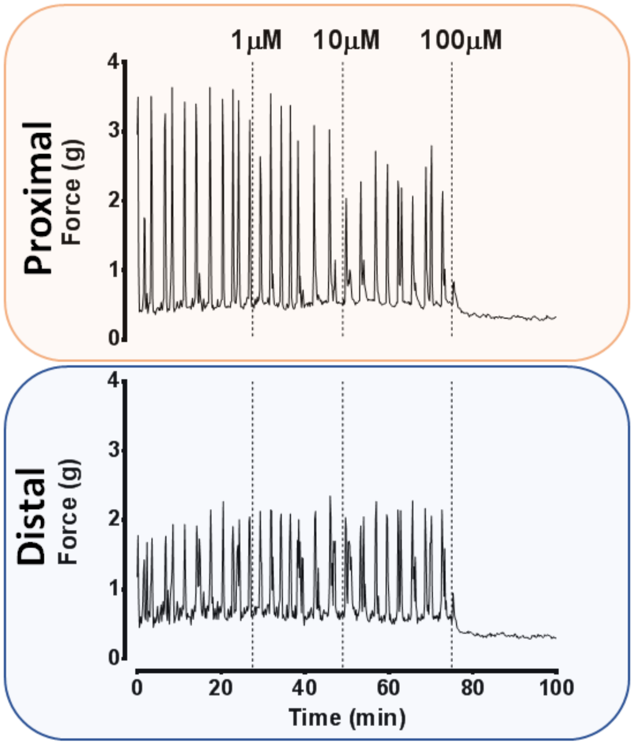

Supplementary Figure 4. Effect of LPA on CMC activity in mice. Representative examples of raw traces from proximal and distal colon recording sites in organ bath experiments where colonic contractions were measured during CMC activity. LPA attenuates the magnitude of colonic contractions in a concentration-dependent manner and this effect is most pronounced in the proximal colon. Traces are representative of n = 6 mice.

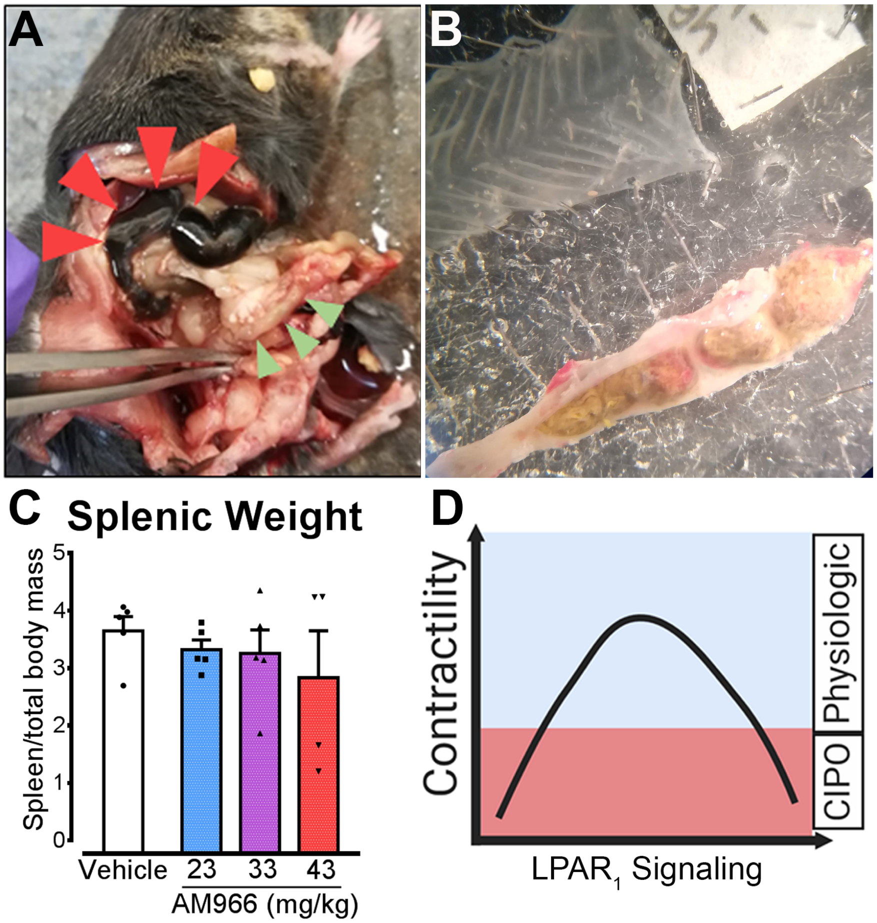

<u>Supplementary Figure 5</u>. Effect of AM966 on fecal pellet size. (**A**) Representative example of necrosis in the small bowel (red arrowheads) in a mouse treated with 43mg/kg AM966. Colonic tissue was not grossly affected (green arrowheads). (**B**) Representative photograph depicting markedly enlarged fecal pellets found impacted within the colon of a mouse treated with high dose AM966. The colon was grossly distended and erythematous. In both high- and medium-dose treated animals, fecal pellets were qualitatively inconsistent in length and girth. (**C**) Sub-chronic treatment with ip AM966 did not result in changes in splenic mass, which was normalized to the final body weight of each mouse at the time of death. This suggests that the overall effects, including reductions in body weight, were not likely to be driven by a broad systemic inflammatory response. (**D**) Schematic illustrating the potential balance of $LPAR_1$ signaling in gut motility.

Excessive activation or blockade of $LPAR_1$ is sufficient to alter several different parameters of GI motility. Observations and data representative of n = 4-5 mice, by One-Way ANOVA (**C**).

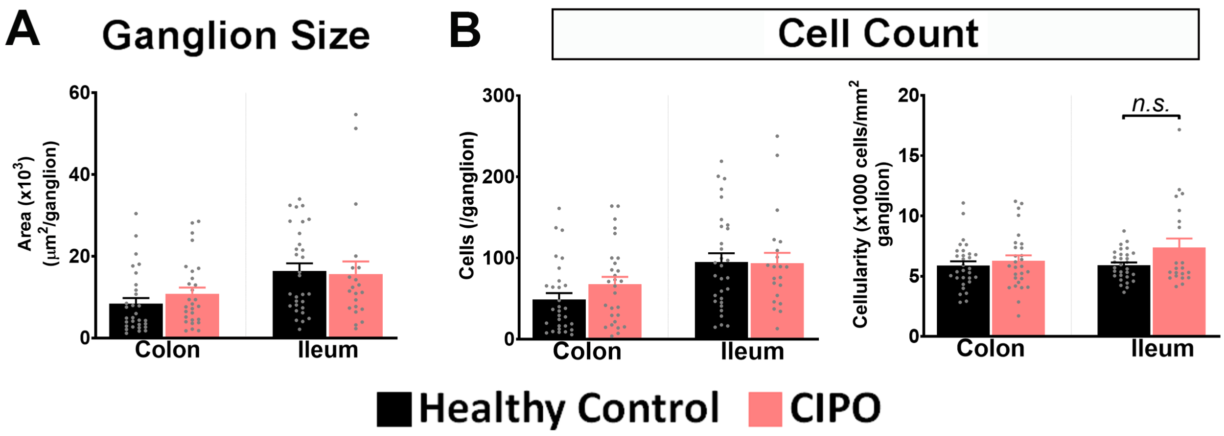

<u>Supplementary Figure 6</u>. Morphological characteristics of human ileum and colon. (**A**) The cross-sectional area of the myenteric plexus is not different between CIPO and healthy controls. This pattern is true in both the ileum and colon. (**B**) Ganglionic cellularity, measured by quantifying the number of cells within a given ganglion (left panel) or number of cells per mm^2 ganglionic area (*right panel*) provided two distinctive measures showing no difference between the ganglionic cellularity observed in the ENS of healthy individuals and patients with CIPO. Data representative of n = 4-6 samples, by one-way ANOVA.

Supplementary Materials and Methods

Human sample collection

Full thickness colon (n=6) or ileum (n=5) (one patient had both ileum and colon) tissue was obtained from adults (n=10; 8 females; age range: 22-73 years) with a diagnosis of CIPO established via clinical assessment, radiological tests (mainly abdominal CT scan) and small bowel manometry at St. Orsola-Malpighi Hospital from 2014 to 2019 (see below section: *Clinical features of patients* for further details).

Comparable tissue samples (n=4 ileum; n=4 colon), obtained from n=4 patients (2 females; age range: 48-73 years) (asymptomatic for previous GI symptoms and referred to surgery for non-complicated GI tumors) were used as controls.

All tissue specimens were immediately fixed in cold neutral 4% formaldehyde (Kaltek, Padua, Italy), paraffin- embedded, cut into 5-μm-thick sections, and mounted serially on poly-L-lysine-coated slides (Thermo Fisher Scientific, Braunschweig, Germany).

All experimental procedures were approved by the Ethics Committee of St. Orsola-Malpighi Hospital for handling and analysis of tissue samples from patients with severe gut dysmotility (EM/146/2014/O).

Clinical features of patients

Patients with CIPO secondary to infectious, neurological, metabolic, systemic autoimmune, and paraneoplastic conditions as well as cases secondary to known genetic abnormalities were excluded. Thus, all patients included in this study were idiopathic in origin. Anorexia nervosa was also specifically excluded in all patients with malnutrition by a psychiatric examination.

The clinical diagnosis of CIPO was established on the basis of a chronic (>3 months), severe symptom complex mimicking mechanical small bowel obstruction, and (at least on one occasion) radiological evidence of air-fluid levels or dilated small bowel loops.

Prior to laparoscopic surgery for tissue collection, patients completed a clinical questionnaire reporting the following data, symptoms, and signs: age, sex, BMI, onset of symptoms, number of sub-obstructive episodes that occurred prior to surgery, abdominal distension, abdominal pain, nausea, vomiting, fullness, early satiety, constipation, diarrhea, esophageal involvement (i.e. motor abnormalities assessed by standard esophageal manometry), gastroparesis (established by scintigraphic gastric emptying), small intestine bacterial overgrowth (SIBO) (determined by glucose/lactulose breath test), urinary symptoms, and small bowel dilation (detectable at abdominal CT scan or MRI). Medications used by each subject were also recorded. These features were summarized in Table 1.

In 4 patients with CIPO, the ileum was the main site of the disease with at least 3/4 patients reporting symptoms of nausea, vomiting, abdominal fullness, early satiety, diarrhea, or constipation. Of these ileum biopsies, only one derived from a male patient while the remaining three were from female patients. In a separate group of 6 patients with CIPO, the disease was localized to the colon. All patients with colonic CIPO reported experiencing constipation while diarrhea was only reported by one patient. In addition, 4 patients with colonic CIPO reported experiencing mild-to-moderate grade abdominal pain while 1 patient reported experiencing severe abdominal pain. Importantly, the body mass index (BMI) of patients with CIPO affecting the ileum (16.28±1.18, n=4) was lower than that of patients where CIPO affected the colon (20.40±0.39, n=6; p=0.033) suggesting differences in long-term nutritional status between the two groups.

Animal Use

All experiments were conducted according to guidelines established by the National Institutes of Health (NIH) Guide for the Care and Use of Animals. All protocols were subject to rigorous institutional oversight administered by the Michigan State University Institutional Animal Care and Use Committee (IACUC).

Deparaffinization and antigen retrieval

Human tissues were deparaffinized and subjected to heat-induced epitope retrieval using a modified version of techniques described elsewhere(*1*). Briefly, deparaffinization involved fully submerging slides in two changes of xylene followed by one change of 1:1 xylene:ethanol solution. At this stage, slides were submerged for 10min during each submersion step. Tissues were then hydrated by submerging slides in two changes of 100% ethanol for 3 minutes, 95% ethanol for 1 minute, 90% ethanol for 1 minute and 80% ethanol for 1 minute before being gently rinsed in distilled water. Antigen retrieval was performed by submerging slides for 40 mins in a dish containing sodium citrate buffer solution (10mM sodium citrate, 0.05% Tween-20, pH 6.0), which was preheated on a hotplate to a constant temperature of 95-100°C. Samples were then removed and allowed to cool to room temperature before conducting immunohistochemical studies.

Animal models

Transgenic *Wnt1^{Cre};GCaMP5g-tdT* mice generated on a C57/Bl6 background were used to study calcium (Ca^{2+}) responses in the ENS. As described previously, Cre recombinase in Wnt1+ cells of the neural crest drives the expression of the fluorescent Ca^{2+} sensor, GCamp5g, in both neurons and glia while the tdTomato reporter is only expressed in glia. This allows Ca^{2+} responses to be readily distinguished between these two cell populations. For all experiments, male and female animals were used between 10 and 14 wks of age and were housed in a

temperature-regulated facility where they were exposed to a 12 hr light:dark cycle and provided *ad libitum* access to standard chow and water. Mouse genotypes were verified commercially by Transnetyx Inc. (Cordova, TN). For immunohistochemistry and motility-related experiments, WT C57/Bl6 mice were used.

Mouse colonic tissue isolation and processing

Mice were euthanized by cervical dislocation followed by decapitation in accordance with guidelines established by the Michigan State University institutional ethics policy. Fresh colon tissue was collected in cold Dulbecco's Modified Eagle Medium (DMEM) before being further processed for use in Ca^{2+} imaging, immunohistochemistry, or colonic migrating motor complex (CMMC) studies. For Ca^{2+} imaging studies only, the DMEM solution was supplemented with 3 μM nicardipine to paralyze the muscular apparatus and enable recording of ENS responses.

Tissue dissection and fixation

Isolated colonic tissue was longitudinally slit open along the mesenteric border and pinned out using insect pins in a 35 mm dish coated with Sylgard™. The tissue was then fixed with Zamboni's fixative overnight at 4°C for subsequent immunohistochemical studies. 4% paraformaldehyde (4% PFA) was used instead for RNAscope. For Ca^{2+} imaging studies, the epithelium was removed and whole-mount circular muscle-myenteric plexus (CMMP) preparations were generated by flipping the tissue and removing the longitudinal muscle layer by microdissection(*2*). In the resultant preparation, the myenteric plexus maintains its structural integrity as the thick circular muscle layer is not damaged.

Immunohistochemistry

Following overnight fixation in Zamboni's buffer or 4% PFA, mouse colonic tissue was further micro-dissected according to techniques described previously. In brief, the epithelium was removed along with the circular smooth muscle layer thereby generating a longitudinal muscle-myenteric plexus (LMMP) preparation. At this point, a 1x1 cm segment of tissue was then cut out and washed three times in phosphate buffered saline (PBS) containing 0.1% Triton X-100 (PBST). For both human and fixed mouse tissues, samples were incubated for 40 mins with a blocking solution containing 4% normal goat serum, 0.4% Triton X-100 and 1% bovine serum albumin dissolved in PBS. Tissues were then incubated overnight at 4°C with the following antibodies: chicken anti-GFAP (1:1000; Cat#ab4674, Abcam, Cambridge, UK), rabbit anti-LPAR1 (1:250; Cat#ab23698, Abcam, Cambridge, UK)(*3*), guinea-pig anti-PGP9.5 (1:500; Cat#GP14104, Neuromics, Edina, MN), biotinylated anti-HuC/D (1:200; Cat#A21272, Invitrogen, Carlsbad, CA), mouse anti-peripherin (1:100; Cat# sc-377093, Santa Cruz Biotechnology, Santa Cruz, CA), rabbit anti-S100β (1:200; Cat#ab52641, Abcam, Cambridge, UK) or rabbit anti-doublecortin (1:200; Cat#ab18723, Abcam, Cambridge, UK). On the day of imaging, samples were incubated with the following fluorescently-conjugated secondary antibodies for 2 h: Alexa 488 donkey anti-chicken (Cat#703-545-155, Jackson, Westgrove, PA), Alexa 488 donkey anti-rabbit (Cat#711-545-152; Jackson, Westgrove, PA), Alexa 594 donkey anti-rabbit (Cat#711-585-152; Jackson, Westgrove, PA), Alexa 594 donkey anti-guinea pig (Cat#706-585-148; Jackson, Westgrove, PA), Alexa 594 goat anti-chicken (Cat#103-585-155; Jackson, Westgrove, PA), streptavidin-Alexa 594 (Cat#016-580-084; Jackson, Westgrove, PA), streptavidin-Alexa 488 (Cat#016-540-084; Jackson, Westgrove, PA), Alexa 647 donkey anti-rabbit (Cat#711-605-152; Jackson, Westgrove, PA), donkey anti-chicken Cy5 (Cat#703-175-155; Jackson, Westgrove, PA) and/or streptavidin Dylight Alexa 405 (Cat# 016-470-084; Jackson, Westgrove, PA). All secondary antibodies were used at a dilution of 1:400. Prior to imaging, tissues were mounted in 4',6-diamidino-2-phenylindole (DAPI) fluoromount G (Cat#0100-20; Southern Biotech, Birmingham, AL).

The specificity of rabbit anti-LPAR1 antibody has been confirmed elsewhere(*3*) but was further demonstrated here by conducting a pre-adsorption assay between the primary antibody and its immunizing peptide sequence (Cat#10006984, Cayman, Ann Arbor, MI) according to manufacturer instructions. Briefly, this entailed incubating the anti-LPAR1 antibody with the immunizing peptide (GGYLPFRDPNSEENSNDIAL) in a 1:1 ratio (v/v) for 1 h at room temperature with occasional mixing. The antibody-peptide cocktail was then diluted to the usual 1:250 antibody dilution and the remaining protocol proceeded as previously described.

Fluorescence in situ hybridization (RNAscope)

Colonic LMMP tissue for RNAscope was generated following fixation and dissection procedures described above (see sections: tissue dissection and fixation and immunohistochemistry). RNAscope was performed using the Advanced Cell Diagnostics RNAscope 2.5 HD Assay—RED (ACD, Cat#322350) according to manufacturer instructions with adjustments for colonic LMMP tissue. Briefly, tissue was dehydrated and subsequently rehydrated by a serial ethanol gradient (25%, 50%, 75%, 100% in PBST) before H_2O_2 treatment. Tissue was then digested with Protease III for 45 mins and incubated with probes for *Lpar1* (ACD, Cat#318591), DapB (ACD, Cat#310043), Ppib (ACD, Cat#313911), Ret (ACD, Cat#431791) or Sox10 (ACD, Cat#435931) overnight at 40°C. All RNAscope steps were performed in a 96-well plate while wash steps were performed in a 48-well plate for 3x5 mins. Following completion of the RNAscope protocol, immunohistochemistry and tissue mounting were performed as described above.

Ca^{2+}-imaging studies

Ca^{2+} imaging studies were conducted in CMMP preparations(*2*), which were perfused with Kreb's buffer (37°C) at a constant rate (2–3 mL/min). The perfusate was removed using a Peri-

Star Pro peristaltic pump (World Precision Instruments Inc.). Unless otherwise specified, all imaging studies were conducted on an upright Olympus BX51WI fixed-stage microscope (Center Valley, PA). Ganglia were viewed at 20x through a wide-field water-immersion objective lens (Olympus XLUMPLFLN20xW, 1.0 numerical aperture). The fluorescent tdTomato reporter was highly expressed in resident glial cells. Illumination for fluorescence imaging was provided by a DG4 Xenon light source (Sutter Instrument, Novato, CA).

GCamp5g photoexcitation was filtered through a 485/20-nm band-pass filter, and emitted light was filtered through a 515-nm long-pass filter. tdT signal was excited by light filtered through a 535/20-nm band-pass filter and reflected tdT signals were filtered through a 610/75-nm band-pass emission filter(*4*). Imaging data were acquired at a frame rate of 0.2 - 1 frames per second using a Neo sCMOS camera (Andor, South Windsor, CT). In some cases, confocal video fluorimetry was performed using a confocal spinning-disk microscope (Nikon A1R HD25; Nikon, Tokyo, Japan), providing higher spatiotemporal resolution of Ca^{2+} responses, which were imaged through a 20x Nikon objective lens (CFI Apochromat LWD Lambda20xC WI, 0.95 numerical aperture). Confocal images were captured using a Nikon DS-Ri2 digital camera (Nikon, Tokyo, Japan) and recorded using NIS-Elements C software (Nikon). For epifluorescence and confocal fluorescence imaging studies, all data were saved on a personal computer running Windows 10 (Microsoft Corporation, WA) and MetaMorph (Molecular Devices, Sunnyvale, CA) and exported as *.tiff* stacks for analysis with Fiji software (NIH)(*5*). To inhibit glial metabolism, some CMMP whole mounts were incubated with FA (5 mM dissolved in DMEM; 37°C; 5% CO2, 95% air) for 2 h before tissue imaging as previously described.

Electrical field stimulation

Electrical field stimulation (EFS) was used to activate neurons using electricity. This was accomplished by applying depolarizing pulses to the tissue using two platinum wires using a

GRASS S9E Electrical Stimulator (Quincy, MA) with the parameters: +70V, 10Hz and 0.1ms duration.

Local drug application

ADP or 18:1 LPA were applied locally to the ganglion surface to provoke agonist responses. To do this, glass capillary applicators were fabricated with a pipette puller (P-87 Flaming-Brown Micropipette Puller, Sutter Instruments Corporation, Novato, CA, USA) and back-filled with drug, dissolved in Kreb's buffer. Drugs were then applied using very gentle positive pressure that was applied with a 1-mL syringe connected to a pipette holder. This approach delivered picoliter volumes of drug and only affected the ganglion within the field of view. We confirmed that the shear fluid stress associated with drug application did not activate neurons or glia under these conditions (not shown).

Solutions and chemicals

DMEM was obtained from Life Technologies (Carlsbad, CA). Unless otherwise noted, all reagents were obtained from Sigma (St. Louis, MO). The composition of Kreb's solution used during imaging experiments was as follows: 121 mM Na^+, 4.9 mM K^+, 25 mM $NaCO_3^-$, 1.2 mM Mg^{2+}, 2.5 mM $CaCl_2$, 1.2 mM $NaHPO_3^-$ 11 mM D-glucose, and 1 mM pyruvate. Kreb's solution was titrated to pH 7.4 with NaOH. 1-oleoyl-2-hydroxy-sn-glycero-3-phosphate (18:1 LPA; Avanti Polar Lipids, Inc., Alabaster, AL) was dissolved in 50% ethanol (v/v) according to manufacturer instructions, and care was taken to avoid surpassing its critical micelle concentration(*6*). AM966 (Cayman Chemical Company, Ann Arbor, MI; Cat#22048) was dissolved in DMSO to a final stock concentration of 50 mM.

Data analysis and statistics

All analyses were conducted in Fiji(*5*). Briefly, video analyses were conducted after background-subtracting recordings, which were first stabilized using the StackReg plugin (http://bigwww.epfl.ch/thevenaz/stackreg/). The tdT channel imaged at each experiment's outset served to identity the location of glial cells and was used to generate corresponding regions of interest (ROIs). Neuronal ROIs were identified by exclusion of glial ROIs, and manually selected based on morphological features. All glial and neuronal ROIs were saved. Ca^{2+} responses were measured using these ROIs and exported to Microsoft Excel (Microsoft Corporation, WA). Unless otherwise indicated, values are reported as fold-change in mean cellular fluorescence intensity relative to baseline florescence intensity ($\Delta F/F_o$) ± standard error of the mean (SEM). For Ca^{2+} imaging studies, sample size denotes the number of cells responding under those experimental conditions with the number of mice utilized is included where appropriate. For all experiments, approximately 30–40 neurons and a comparable number of glia were studied per ganglion, with at least 1–2 ganglia utilized per mouse under each recording condition. For IHC experiments, protein expression in the human and mouse ENS was computed by normalizing cross-sectional protein abundance to total ganglionic area and/or net ganglionic cell count. Grubb's test was used to compute statistical outliers, which were excluded from the final analysis only if the confidence threshold of α=0.05 was surpassed. In most instances, statistical testing involved unpaired two-tailed Student's *t*-tests comparing $\Delta F/F_o$ values between different conditions. Welch's correction was applied as needed when data variability was unequal between the groups being compared. Final figures were produced with GraphPad Prism 5 (GraphPad Software Inc., La Jolla, CA), and custom illustrations were designed in BioRender software (https://biorender.com/).

AM966 injections and carmine transit studies

Age-matched males and females were randomly assigned to one of 4 possible treatment groups: low-dose AM966 (22 mg/kg), medium-dose AM966 (33 mg/kg), high-dose AM966 (43 mg/kg) or vehicle(*7*). WT C57/Bl6 mice were then injected intraperitoneally with the $LPAR_1$

antagonist, AM966 (Cat#22048; Cayman, Ann Arbor, MI) q24 for 3 days, or vehicle. The vehicle control consisted of DMSO dissolved in sunflower oil which at most delivered a DMSO dosage far below the 10 ml/kg upper limit reported for mice(*8*). Mouse weights were measured longitudinally over the duration of the experiment, beginning on the first day of injections. Drug dosages were selected based on previous *in vivo* reports of the systemic effects and pharmacokinetic properties of AM966 in mice as well as *in vitro* studies on the binding affinity of AM966 at the LPA1 receptor.

After 3-days of continuous exposure to AM966, the *in vivo* effect of sustained, sub-chronic $LPAR_1$ blockade was assessed by determining the whole-bowel transit of Carmine red dye. As described elsewhere (*9-11*), mice were administered 200 μL of 6% Carmine red solution in water supplemented with 0.5% methylcellulose by oral gavage. After 1h, mice were euthanized, and the entire GI tract was carefully but rapidly removed and laid out at neutral length on an all-white surface. The Carmine red wavefront was then grossly visualized within the gut lumen as a discrete, bright-red bolus that was immediately trailed by a dye-free bowel segment, and its distance relative to the gastric fundus was measured. Additional parameters that were noted included total bowel length, splenic weight, location of the ileocecal pouch and the presence of strictures or serosal hemorrhages. The colonic regions of these tissue were then collected in drug-free DMEM for subsequent fixation and further immunohistochemical characterization of ENS architecture.

Colonic motor complexes (CMCs)

Colonic motility was assessed in response to $LPAR_1$ receptor activation ex vivo. Briefly, whole intact colon was rapidly collected in drug-free DMEM/F-12 maintained at 37°C. The oral and aboral ends of the colon were then mounted in place on a stainless-steel holding rod after lumen contents were flushed. Two force transducers (Grass Instruments) were connected to the gut wall via surgical silk approximately 2cm apart. The baseline tension was calibrated to 0.5 g

and spontaneous CMC production was monitored for a period of 20 min before experiments were conducted. The last 5 min of this acclimatization period was used as a baseline for analysis, which was conducted in LabChart 8 (ADInstruments, Colorado Springs, CO). Drugs were added cumulatively to the bath and the resultant changes in CMC amplitude and integral were measured in relation to baseline.

Supplemental References

1. S. R. Shi, B. Chaiwun, L. Young, R. J. Cote, C. R. Taylor. Antigen retrieval technique utilizing citrate buffer or urea solution for immunohistochemical demonstration of androgen receptor in formalin-fixed paraffin sections. *J. Histochem. Cytochem.* **41**, 1599–1604 (1993).

2. N. J. Spencer, E. J. Dickson, G. W. Hennig, T. K. Smith. Sensory elements within the circular muscle are essential for mechanotransduction of ongoing peristaltic reflex activity in guinea-pig distal colon. *J. Physiol.* **576**, 519–531 (2006).

3. E. Liszewska *et al.* Lysophosphatidic acid signaling during embryo development in sheep: Involvement in prostaglandin synthesis. *Endocrinology*. **150**, 422 (2009).

4. I. A. M. Brown, J. L. McClain, R. E. Watson, B. A. Patel, B. D. Gulbransen. Enteric glia mediate neuron death in colitis through purinergic pathways that require connexin-43 and nitric oxide. *CMGH*. **2**, 77–91 (2016).

5. J. Schindelin *et al.*, Fiji: An open-source platform for biological-image analysis. *Nat. Methods*. **9** (2012), pp. 676–682.

6. Z. Li, E. Mintzer, R. Bittman. The critical micelle concentrations of lysophosphatidic acid and sphingosylphosphorylcholine. *Chem. Phys. Lipids*. **130**, 197–201 (2004).

7. J. S. Swaney *et al.* A novel, orally active LPA(1) receptor antagonist inhibits lung fibrosis in the mouse bleomycin model. *Br. J. Pharmacol.* **160**, 1699–1713 (2010).

8. S. C. Gad, C. D. Cassidy, N. Aubert, B. Spainhour, H. Robbe. Nonclinical vehicle use in studies by multiple routes in multiple species. *Int. J. Toxicol.* **25**, 499–521 (2006).

9. J. L. McClain, D. E. Fried, B. D. Gulbransen. Agonist-evoked Ca2+ signaling in enteric glia drives neural programs that regulate intestinal motility in mice. *Cmgh*. **1**, 631–645 (2015).

10. J.P. White, S. Xiong, N.P. Malvin, W. Khoury-Hanold, R.O. Heuckeroth, T.S. Stappenbeck, M.S. Diamond. Intestinal dysmotility syndromes following systemic infection by flaviviruses. *Cell.* **175**(5):1198-1212 (2018).

11. N. Dey, V.E. Wagner, L.V. Blaton, J. Cheng, L. Fontana, R. Haque, T. Ahmed, J.I. Gordon. Regulators of gut motility revealed by a gnotobiotic model of diet-microbiome interactions related to travel. *Cell.* **163**(1):95-107 (2015).
